# Upregulation of Superenhancer‐Driven LncRNA FASRL by USF1 Promotes De Novo Fatty Acid Biosynthesis to Exacerbate Hepatocellular Carcinoma

**DOI:** 10.1002/advs.202204711

**Published:** 2022-10-28

**Authors:** Jiang‐Yun Peng, Dian‐Kui Cai, Ren‐Li Zeng, Chao‐Yang Zhang, Guan‐Cheng Li, Si‐Fan Chen, Xiao‐Qing Yuan, Li Peng

**Affiliations:** ^1^ Guangdong Provincial Key Laboratory of Malignant Tumor Epigenetics and Gene Regulation Guangdong‐Hong Kong Joint Laboratory for RNA Medicine Sun Yat‐sen Memorial Hospital Sun Yat‐sen University Guangzhou 510120 P. R. China; ^2^ Medical Research Center Sun Yat‐sen Memorial Hospital Sun Yat‐sen University Guangzhou 510120 P. R. China; ^3^ Department of Hepatobiliary Surgery Sun Yat‐sen Memorial Hospital Sun Yat‐sen University Guangzhou 510120 P. R. China; ^4^ Department of Endocrinology Sun Yat‐sen Memorial Hospital Sun Yat‐sen University Guangzhou 510120 P. R. China; ^5^ Division of Functional Genome Analysis German Cancer Research Center (DKFZ) 69120 Heidelberg Germany; ^6^ Key Laboratory of Carcinogenesis of the Chinese Ministry of Health and the Key Laboratory of Carcinogenesis and Cancer Invasion of Chinese Ministry of Education Central South University Changsha 410078 P. R. China; ^7^ Cancer Research Institute Central South University Changsha 410078 P. R. China; ^8^ Breast Tumor Center Sun Yat‐sen Memorial Hospital Sun Yat‐sen University Guangzhou 510120 P. R. China

**Keywords:** acetyl‐CoA carboxylase 1, fatty acid synthesis, fatty acid synthesis‐related long noncoding RNAs, hepatocellular carcinoma, lipid metabolism, long noncoding RNA, superenhancer

## Abstract

Superenhancers drive abnormal gene expression in tumors and promote malignancy. However, the relationship between superenhancer‐associated long noncoding RNA (lncRNA) and abnormal metabolism is unknown. This study identifies a novel lncRNA, fatty acid synthesis‐related lncRNA (FASRL), whose expression is driven by upstream stimulatory factor 1 (USF1) through its superenhancer. FASRL promotes hepatocellular carcinoma (HCC) cell proliferation in vitro and in vivo. Furthermore, FASRL binds to acetyl‐CoA carboxylase 1 (ACACA), a fatty acid synthesis rate‐limiting enzyme, increasing fatty acid synthesis via the fatty acid metabolism pathway. Moreover, the expression of FASRL, USF1, and ACACA is increased, and their high expression indicates a worse prognosis in HCC patients. In summary, USF1 drives FASRL transcription via a superenhancer. FASRL binding to ACACA increases fatty acid synthesis and lipid accumulation to mechanistically exacerbate HCC. FASRL may serve as a novel prognostic marker and treatment target in HCC.

## Graphical abstract

This study identifies a novel lncRNA, fatty acid synthesis‐related lncRNA (FASRL), whose expression is driven by upstream stimulatory factor 1 through a superenhancer. FASRL binding to acetyl‐CoA carboxylase 1 increases fatty acid synthesis and lipid accumulation to mechanistically exacerbate hepatocellular carcinoma (HCC). FASRL serves as a novel potential prognostic marker and treatment target in HCC.

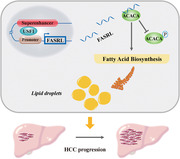

## 1 Introduction

Liver cancer is one of the most common cancers, and ≈906 000 new cases and 830 000 related deaths were recorded in 2020; liver cancer is the sixth most commonly diagnosed cancer and the third highest cause of cancer‐related death worldwide.^[^ ^1^ ^]^ It is estimated that the incidence of liver cancer will exceed 1 million by 2025, resulting in a very serious global health challenge.^[^ ^2^ ^]^ Hepatocellular carcinoma (HCC) is the most common type of liver cancer, accounting for ≈90% of all liver cancer cases. There are many factors that cause HCC, mainly including hepatitis B virus or hepatitis C virus infection, nonalcoholic steatohepatitis, excessive alcohol consumption, type 2 diabetes, and smoking.^[^ ^2^ ^]^ Although therapeutic treatments for HCC have improved with the advancement of medicine, such as the several oral tyrosine kinase inhibitors that have been approved by the U.S. Food and Drug Administration for the treatment of advanced HCC, the therapeutic benefits are still limited,^[^ ^3^ ^]^ and the five‐year survival rate of HCC patients remains bleak. Moreover, the pathological development of HCC is a complex and multistep process. Our previous research confirmed that the long noncoding RNA (lncRNA) HCCL5 promotes the progression of HCC via the epithelial–mesenchymal transition (EMT).^[^ ^4^ ^]^ Further studies of the pathogenesis and therapeutic targets for HCC are urgently needed.

There are many ways to regulate tumor progression, and metabolic changes are important regulatory factors. Lipid metabolism dysregulation is one of the most prominent metabolic phenotypes in cancers. Increased lipid synthesis or uptake promotes the rapid growth of cancer cells and induces tumor formation.^[^ ^5^ ^]^ In colorectal adenocarcinoma, breast cancer, and esophageal adenocarcinoma, an increase in the contents of fatty acids (FAs) or lipids in tumor cells can exacerbate tumor progression.^[^ ^6^ ^]^ Lipids are hydrophobic molecules and include sterols, monoglycerides, diglycerides, triglycerides, phospholipids, and glycolipids. Many lipids are derived from FAs, which can be classified according to their length (number of carbon atoms) and saturation (number of double bonds). FAs can be produced through de novo biosynthesis. By limiting the use of FAs in tumors, tumor cell growth can be controlled to inhibit tumor progression.^[^ ^7^ ^]^ Furthermore, FA metabolism is indispensable in HCC. Specifically, an increase in the accumulation of FAs in tumor cells promotes the progression of HCC by promoting FA synthesis or inhibiting FA oxidation.^[^ ^8^ ^]^ In contrast, the inhibition of FA metabolism restricts the proliferation of HCC.^[^ ^9^ ^]^ However, knowledge regarding the epigenomic regulatory network related to the regulation of FA metabolism in HCC is limited, and further research is needed to elucidate it.

Although less than 2% of the human genome encodes proteins, most nucleotides in the genome can be transcribed, resulting in large amounts of non‐coding RNA transcripts.^[^ ^10^ ^]^ This class of RNAs includes lncRNAs, which are transcripts of more than 200 nucleotides in length.^[^ ^11^ ^]^ Similar to mRNAs, lncRNAs are transcribed by RNA polymerase II, and this transcriptional activity can be regulated by *cis*‐acting elements like enhancers and superenhancers, and by *trans*‐acting factors like transcription factors (TFs).^[^ ^4^, ^12^ ^]^ The regulation of lncRNA expression in tumors is delicate and complex, and lncRNAs participate in many key cancer phenotypes by interacting with biological macromolecules, such as DNAs, mRNAs, and proteins, thus playing a regulatory role in the occurrence and development of tumors.^[^ ^11^, ^13^ ^]^ However, compared with mRNAs, lncRNAs have a relatively low expression, alternative forms of biogenesis, complex structural characteristics, and noncoding features, and they generally lack conservation, thus, most lncRNA functions have not yet been investigated.^[^ ^14^ ^]^ In addition, the specific epigenomic regulatory network of lncRNAs, including superenhancers, is largely unclear.

In this work, we chose HCC as a disease model, and aimed to clarify the specific mechanism by which superenhancer‐related lncRNAs function in lipid metabolism. We discovered a novel cancer‐promoting lncRNA, FASRL, whose expression is regulated by a dysregulated superenhancer and that can promote the de novo synthesis of FAs to promote HCC. This study helps to further elucidate the pathogenesis of HCC, and provides a new perspective for prognosis prediction and targeted treatments for HCC.

## 2 Results

### 2.1 A Novel Superenhancer‐Related LncRNA, FASRL, Was Identified in HCC

**Figure 1:**
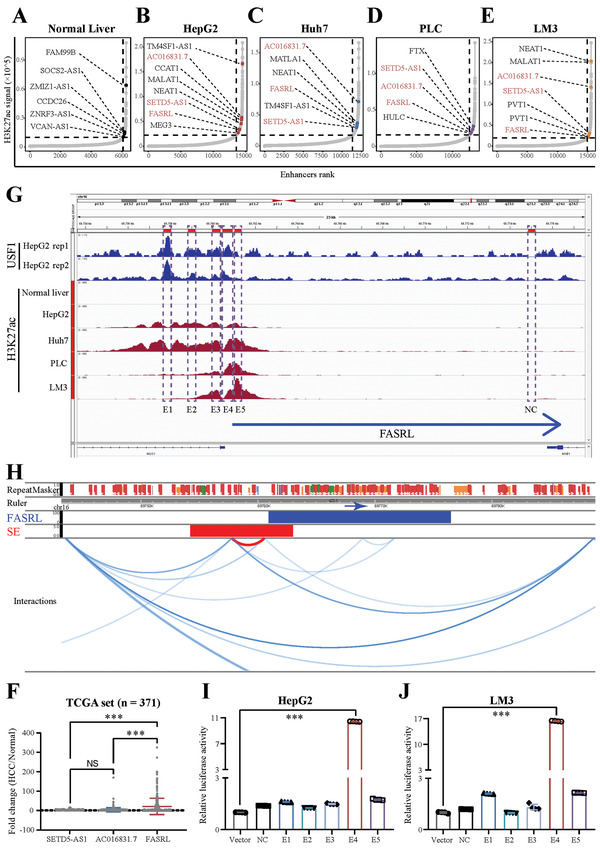
A novel superenhancer‐related lncRNA, FASRL, was identified in HCC. Hockey stick plot showing superenhancer‐related lncRNAs in the A) normal liver and B) HepG2, C) Huh7, D) PLC, and E) LM3 cell lines. The colored dots on the curve indicate some classic and novel lncRNAs in the normal liver and four HCC cell lines. F) The scatter plot showing the fold change in the expression of three lncRNAs in HCC samples compared with normal liver tissue samples. The expression of three lncRNAs in HCC tissues and adjacent normal liver tissues was downloaded from TCGA website. *n* for HCC = 371, *n* for Normal = 50; fold change, the ratio of the lncRNA expression in HCC tissues to the mean of lncRNA expression in normal liver tissues. G) ChIP‐seq profiles of USF1 and H3K27ac in normal liver, HepG2, Huh7, PLC, and LM3 cell lines. NC and E1‐E5 represent the genomic positions of the NC sequence and five enhancer sequences, respectively. The blue arrow indicates the FASRL transcription direction. H) Chromatin interaction analysis by paired end labeling (ChIA‐PET) data analysis revealed the spatial interaction between the superenhancer and FASRL promoter on chromatin. SE, superenhancer. The original sequencing data from a ChIA‐PET in the HepG2 cell line were downloaded from the ENCODE database. The dual‐luciferase experiment showing the transcriptional activity of NC and E1‐E5 in the I) HepG2 and J) LM3 cell lines. The data are expressed as the mean ± SD. NS, non‐significance; ***p < 0.001.

Superenhancers (SEs) specifically drive the expression of genes including lncRNAs, and superenhancer‐driven gene expression is an important mechanism by which tumors are exacerbated. Here, we screened and identified lncRNAs that are regulated by aberrant superenhancers in HCC cells. As expected, many common tumor‐related lncRNAs, such as MALAT1, NEAT1, HULC, and PVT1, were identified in the H3K27ac chromatin immunoprecipitation sequencing (ChIP‐seq) data (**Figure** **1**A–E). Importantly, we identified 31 superenhancer‐associated lncRNAs that were shared in four HCC cell lines but not observed in normal liver tissue according to the ChIP‐seq results (Figure S1A, Supporting Information). Three of them were upregulated in HCC samples, and their high expression was correlated with a worse prognosis in HCC patients (Figure S1B, Supporting Information). We selected one of the three lncRNAs for subsequent study because it exhibited the largest fold change in expression in HCC patient samples compared with the other two lncRNAs (Figure 1F). Of note, SETD5‐AS1 has been previously reported,^[^ ^15^ ^]^ and the other two are novel lncRNAs, and the selected lncRNA knockdown inhibited HCC cell proliferation more than SETD5‐AS1 knockdown in a Cell Counting Kit‐8 (CCK‐8) assay in vitro (Figure S2A–D, Supporting Information). We named this lncRNA “FASRL” on the basis of the subsequent results. FASRL is located on chromosome 16, has a length of 15551 bp, and contains two exons and one intron (Figure S3A, Supporting Information). The metabolic activity of HepG2 cell line is relatively stable, and usually used as a cell model in metabolic studies.^[^ ^16^ ^]^ Considering the relatively higher expression of FASRL and the purpose of exploring metabolism, we selected the HepG2 and LM3 cell lines to perform the following experiments (Figure S3B, Supporting Information).

The integrative genomics viewer (IGV)‐visualized ChIP‐seq data showed that a superenhancer peak was present upstream of the FASRL gene in four HCC cell lines but not in normal liver tissues (Figure 1G). The chromatin interaction analysis by paired end labeling (ChIA‐PET) data revealed spatial interactions between the regions of the superenhancer and the promoter of FASRL (Figure 1H). The five enhancer sequences of FASRL were then cloned into the pGL3‐promoter vector for dual‐luciferase experiments to validate the regulation of FASRL expression by these superenhancer constituents. We found that the constituent of superenhancer E4 notably increased the fluorescence intensity in both HCC cell lines (Figure 1I,J). Cyclin‐dependent kinase 7 (CDK7) and bromodomain‐containing protein 4 (BRD4) are two critical components of the trans‐acting superenhancer complex. The CDK7 inhibitor THZ1 and BRD4 inhibitor JQ1 significantly reduced the expression of FASRL in HepG2 and LM3 cell lines (Figure S3C–F, Supporting Information). In summary, we discovered a novel lncRNA, FASRL, whose expression was associated with superenhancer.

### 2.2 The Expression of FASRL Was Driven by Upstream Stimulatory Factor 1 through a Superenhancer

**Figure 2:**
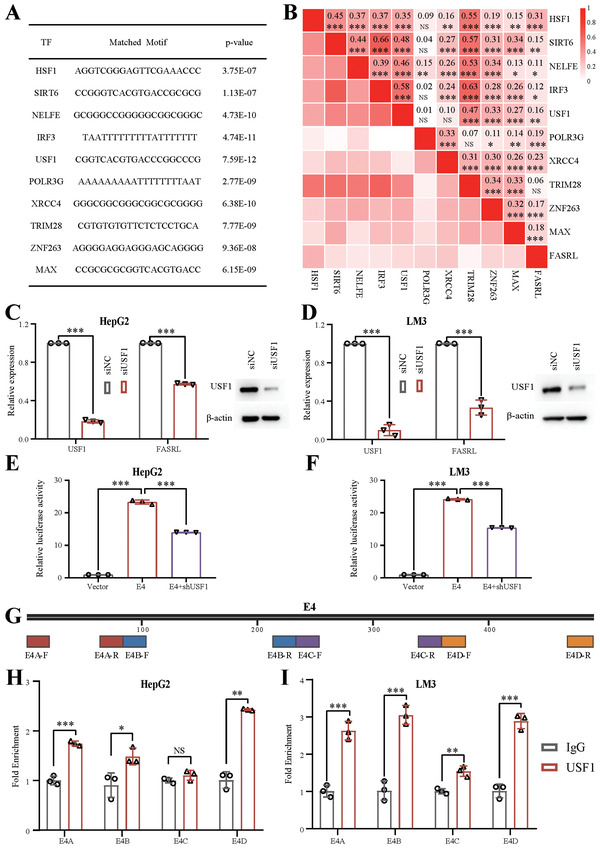
Identification of an upstream TF, USF1, that positively regulated FASRL expression. A) The ten TFs predicted based on the superenhancer sequence of FASRL. B) Correlation analysis of the relative expression of ten TFs and FASRL. The numeric value and color depth represent the correlation coefficient. The expression of ten TFs and FASRL in HCC tissues was downloaded from TCGA website. *n* for HCC = 371. Left, qRT–PCR showing the relative expression of USF1 and FASRL in the C) HepG2 and D) LM3 cell lines after siRNA‐mediated USF1 knockdown; right, Western blot assay showing the protein content of USF1 in the C) HepG2 and D) LM3 cell lines following siRNA‐mediated USF1 knockdown. The dual‐luciferase experiment showing the transcriptional activity of E4 after USF1 expression was knocked down by shRNA in the E) HepG2 and F) LM3 cell lines. G) The four ChIP–qPCR primer pairs designed on the E4 sequence. ChIP–qPCR analysis of the interaction between USF1 and the components of the superenhancer in the H) HepG2 and I) LM3 cell lines. The data are expressed as the mean ± SD. NS, non‐significance; *p < 0.05; **p < 0.01; ***p < 0.001.

To elucidate the upstream mechanism by which the superenhancer regulated FASRL expression, we first performed the motif analysis based on the sequence of the superenhancer (**Figure** **2**A). The top ten TFs based on the motif and correlation analyses were heat shock transcription factor 1 (HSF1), sirtuin 6 (SIRT6), negative elongation factor complex member E (NEFLE), interferon regulatory factor 3 (IRF3), upstream stimulatory factor 1 (USF1), RNA polymerase III subunit G (POLR3G), X‐ray repair cross complementing 4 (XRCC4), tripartite motif‐containing 28 (TRIM28), zinc finger protein 263 (ZNF263), and MYC‐associated factor X (MAX). The correlation between the expression of all ten TFs and FASRL is shown in Figure 2B. Subsequently, we used small interfering RNA (siRNA) pools to respectively knock down the expression of these ten TFs (Figure 2C,D and Figure S4A,B, Supporting Information), and found that knocking down USF1 expression, but not that of the other nine TFs, downregulated the expression of FASRL in both HepG2 and LM3 cell lines (Figure 2C,D and Figure S4C,D, Supporting Information). As shown in the dual‐luciferase experiment, the increase in the fluorescence intensity of E4 was significantly reduced by USF1 shRNA in both the HepG2 and LM3 cell lines (Figure 2E,F and Figure S5A,B, Supporting Information). Furthermore, E4 was divided into four fragments for ChIP–qPCR (Figure 2G), and we observed that USF1 could bind to the E4 region of FASRL (Figure 2H,I). Finally, we conducted ChIP–qPCR of H3K27ac and USF1 in HCC patient tissues (Figure S6A,B, Supporting Information), which was consistent with the results in the HCC cell lines. In summary, the TF USF1 can transcriptionally drive FASRL expression by binding to its superenhancer in HCC.

### 2.3 FASRL Exacerbated the Malignant Phenotype of HCC In Vitro

Next, we explored the function of FASRL in HCC cells in vitro. We first investigated the subcellular localization of FASRL and found that it was present in both the cell nucleus and cytoplasm, and was mainly distributed in the cytoplasm (Figure S7A,B, Supporting Information). Therefore, we designed five different siRNA sequences to target FASRL and examined the knockdown effects of these siRNAs in the HepG2 and LM3 cell lines. We found that siRNA #1 had the best knockdown effect compared with the other sequences. Although siRNA #4 and siRNA #5 both effectively knocked down the expression of FASRL, the knockdown effects of siFASRL#4 in the two liver cancer cell lines were inconsistent; thus, we chose siFASRL#1 and #5 instead of #4 for the subsequent experiments (Figure S8A,B, Supporting Information).

**Figure 3:**
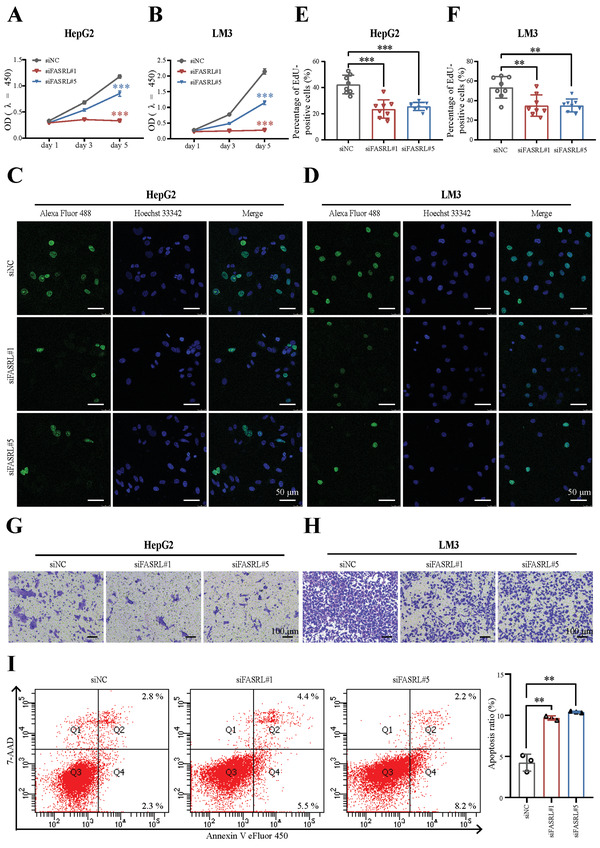
FASRL knockdown inhibited HCC progression in vitro. CCK‐8 assay revealed the growth of the A) HepG2 and B) LM3 cell lines transfected with siFASRL#1, siFASRL#5, and siNC. Representative images of EdU assay revealed the proliferation of the C) HepG2 and D) LM3 cell lines transfected with siFASRL#1, siFASRL#5, and siNC. Quantification of EdU assay in the E) HepG2 and F) LM3 cell lines transfected with siFASRL#1, siFASRL#5, and siNC, *n* = 8 fields of view. Transwell experiment showing the cell migration ability of the G) HepG2 and H) LM3 cell lines transfected with siFASRL#1, siFASRL#5, and siNC. I) Left, apoptosis in the HepG2 cell line transfected with siFASRL#1, siFASRL#5, and siNC was detected by flow cytometry; right, the statistical results of the corresponding apoptotic cells. The data are expressed as the mean ± SD. **p < 0.01; ***p < 0.001.

The CCK‐8 assay showed that the FASRL knockdown significantly inhibited the growth of the HepG2 and LM3 cell lines (**Figure** **3**A,B). Similarly, the EdU experiment showed that the FASRL knockdown significantly suppressed the proliferation ability of HepG2 and LM3 cells (Figure 3C–F). The Transwell experiment showed that knocking down FASRL expression significantly decreased the migration ability of the HepG2 and LM3 cell lines (Figure 3G,H). Moreover, knocking down FASRL expression significantly increased apoptosis of HepG2 cells (Figure 3I). To assess the oncogene properties of superenhancer‐related FASRL, we selected several lncRNAs that were proven to promote HCC progression, including lncRNA PCNAP1,^[^ ^17^ ^]^ lncTCF7,^[^ ^18^ ^]^ TUG1,^[^ ^19^ ^]^ HULC,^[^ ^20^ ^]^ GIHCG,^[^ ^21^ ^]^ and LINC00662,^[^ ^22^ ^]^ to compare the effect of those lncRNAs with that of FASRL on cell proliferation in HCC cell lines. We found that FASRL knockdown led to relatively higher inhibitory effect of cell proliferation in the two HCC cell lines (Figure S9A–D, Supporting Information). It proved that the high‐throughput unbiased screening strategy of FASRL is effective. Then, we performed gain‐of‐function studies by stably overexpressing FASRL in HepG2 and LM3 cell lines (Figure S10A,B, Supporting Information). We found that the overexpression of FASRL significantly accelerated the proliferation and migration of HCC cells in vitro (Figure S10C–H, Supporting Information). To verify these HepG2 and LM3 cell phenotypes in vivo, we constructed cell lines with the stable knockdown of FASRL expression by transfection with a lentivirus carrying shRNA (Figure S11A, Supporting Information). As expected, after knocking down FASRL expression with shRNA in the HepG2 and LM3 cell lines, the proliferation and migration of the HCC cells were significantly inhibited (Figure S11B–D, Supporting Information). These results suggest that FASRL can promote oncogenic growth in HCC cells.

### 2.4 FASRL Promoted the Growth of HCC Xenografts In Vivo

**Figure 4:**
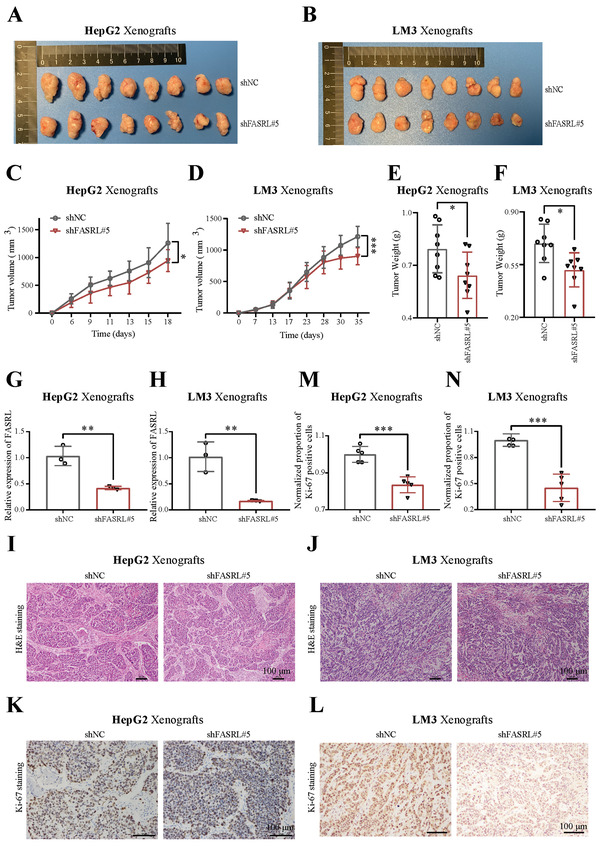
FASRL knockdown decelerated HCC progression in vivo. Representative images showing the shFASRL group and control shNC group of HCC xenografts derived from the A) HepG2 and B) LM3 cell lines. Growth curves of the shFASRL group and shNC group of HCC xenografts derived from the C) HepG2 and D) LM3 cell lines. The weights of the E) HepG2 and F) LM3 cell lines‐derived HCC xenografts in the shFASRL group and shNC group. FASRL expression in the shFASRL group and shNC group of HCC xenografts derived from the G) HepG2 and H) LM3 cell lines, *n* = 3. Representative images of H&E staining of sections of HCC xenografts derived from the I) HepG2 and J) LM3 cell lines. Representative images of Ki‐67 staining of sections of HCC xenografts derived from the K) HepG2 and L) LM3 cell lines. Proportion of Ki‐67‐positive cells in HCC xenografts derived from the M) HepG2 and N) LM3 cell lines, *n* = 5 fields of view. The data are expressed as the mean ± SD. *p < 0.05; **p < 0.01; ***p < 0.001.

The in vitro results confirmed that FASRL can accelerate the tumorigenic phenotype of HCC cell lines, but whether FASRL affects tumor progression in vivo is unknown. Next, HCC cell lines stably transfected with shFASRL or shNC were subcutaneously injected into nude mice. As observed, the HCC tumors in the shFASRL group were smaller than those in the shNC group ( **Figure** **4**A,B). The tumor growth rate in the shFASRL group was obviously slower than that in the shNC group (Figure 4C,D), and the tumor weight in the shFASRL group was lower than that in the shNC group (Figure 4E,F). The survival analysis suggested that the shFASRL group had a relatively longer survival time than the shNC group (Figure S12A,B, Supporting Information). As expected, FASRL expression was downregulated in the xenograft tumor tissues from shFASRL group (Figure 4G,H). Next, we performed H&E staining and immunohistochemistry experiment using tumor tissues collected from nude mice. Lower Ki‐67‐positive rates were observed in the shFASRL group, revealing that shFASRL significantly slowed the proliferation of tumor cells in nude mice (Figure 4I–N). Overall, FASRL knockdown can alleviate the tumor progression of HCC in vivo.

### 2.5 FASRL Upregulated Fatty Acid Synthesis Pathway‐Related Genes in HCC

**Figure 5:**
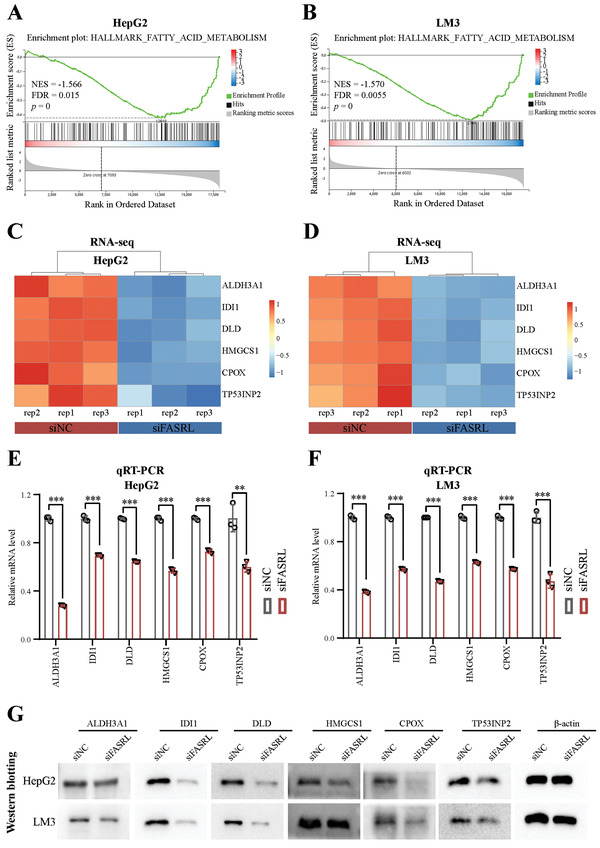
FASRL was involved in the fatty acid metabolism pathway in HCC. GSEA showing that the genes with altered expression after FASRL expression was knocked down in the A) HepG2 and B) LM3 cell lines were enriched in the fatty acid (FA) metabolism pathway. The heatmap showing the genes in the FA metabolism pathway that exhibited significantly altered expression after the knockdown of FASRL expression in the C) HepG2 and D) LM3 cell lines as determined by RNA‐seq. The significantly downregulated mRNA expression of genes in the FA metabolism pathway after the knockdown of FASRL expression in the E) HepG2 and F) LM3 cell lines as measured by qRT–PCR. G) Western blotting showing the altered protein expression of genes in the FA metabolism pathway after FASRL expression was knocked down in the HepG2 and LM3 cell lines. The data are expressed as the mean ± SD. **p < 0.01; ***p < 0.001.

To reveal the specific downstream regulatory pathway of FASRL, we performed RNA‐seq using HCC cell lines after FASRL knockdown. We first validated the knockdown efficiency of FASRL in the HepG2 and LM3 cell lines (Figure S13A,B, Supporting Information). As shown, 636 differentially expressed genes with |log2 foldchange| ≥ 1 and *Q* value < 0.05 were shared in both HCC cell lines (Figure S13C, Supporting Information). The heatmap showed the expression pattern of differentially expressed genes (|log2 foldchange| ≥ 1 and *Q* value < 0.05) in the HepG2 and LM3 cell lines after knocking down FASRL expression (Figure S13D,E, Supporting Information). Furthermore, the gene set enrichment analysis (GSEA) showed that the FASRL knockdown was significantly negatively correlated with the expression of fatty acid (FA) pathway‐related genes in HepG2 and LM3 cells (**Figure** **5**A,B; Tables S1 and S2, Supporting Information). We selected the top‐ranking in foldchange and potential cancer‐promoting genes in the FA metabolism pathway gene set. As shown in volcano plot and heatmap of RNA‐seq data, the expression of these chose genes was decreased in the HCC cells in which FASRL expression was knocked down (Figure 5C,D and Figure S13F,G, Supporting Information). To further validate the RNA‐seq data, qRT‐PCR and Western blot assay were conducted in HCC cells with FASRL knockdown. It was displayed that the mRNA levels (Figure 5E,F) and protein expression of ALDH3A1, IDI1, DLD, HMGCS1, CPOX, and TP53INP2 (Figure 5G) were downregulated in the HepG2 and LM3 cell lines after FASRL expression was knocked down. Our results indicate that the FASRL knockdown downregulated FA metabolism pathway‐related genes in HCC.

### 2.6 FASRL Interacted with Acetyl‐CoA Carboxylase 1, a Key Rate‐Limiting Enzyme in FA Synthesis, and Then Inhibited Its Phosphorylation

**Figure 6:**
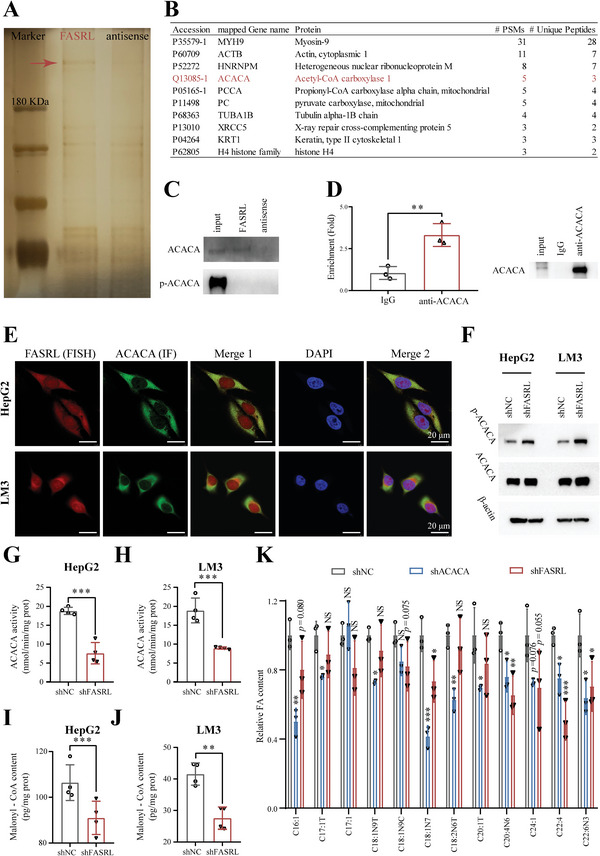
FASRL bound to ACACA and inhibited its phosphorylation, thus promoting FA synthesis in HCC. A) Silver staining of RNA pull‐down fractions. The arrow indicates the specific protein band from the FASRL pull‐down fractions. B) The specific protein in the FASRL pull‐down fractions was identified by mass spectrometry (MS), and the protein marked in red is ACACA. C) Upper panel, Western blotting verified the protein level of ACACA enriched by RNA pull‐down. Bottom panel, Western blotting of p‐ACACA in the input, FASRL, and FASRL antisense samples. D) The RIP experiment showing the binding of ACACA to FASRL by using ACACA antibody (IgG antibody as a control). E) RNA FISH assay of FASRL and immunofluorescence assay of ACACA showing the colocalization of FASRL and ACACA. FISH, fluorescence in situ hybridization; IF, immunofluorescence. Red represents FASRL, green represents ACACA, and merged yellow indicates the colocalization of FASRL and ACACA. F) Western blotting showing the protein contents of ACACA and phosphorylation of ACACA in the HepG2 and LM3 cell lines stably transfected with shFASRL and its control shNC. ACACA enzymatic activity in the shFASRL group and control shNC group in G) HepG2 and H) LM3 cell lines, *n* = 4. Malonyl‐CoA levels in I) HepG2 and J) LM3 cells stably transfected with shFASRL and its control shNC, *n* = 4. K) The contents of fatty acids (FAs) in HepG2 cells stably transfected with shFASRL, shACACA, or shNC. The horizontal axis showing different types of FAs, *n* = 3. The data are expressed as the mean ± SD. NS, non‐significance; *p < 0.05; **p < 0.01; ***p < 0.001.

To further explore the proteins that directly interact with FASRL, we used an RNA pull‐down assay combined with mass spectrometry (MS) to identify RNA‐binding proteins (RBPs) that directly interact with FASRL. Compared with the antisense strand (as a control), RNA pull‐down with the sense strand of FASRL identified a specific band at ≈260 kDa as observed by silver staining (**Figure** **6**A). Next, MS revealed that acetyl‐CoA carboxylase 1 (ACACA) was present in the protein fraction pulled down by the FASRL sense strand, which was consistent with the silver staining result (Figure 6B). This protein was further verified to be ACACA through an RNA pull‐down assay combined with a Western blotting (Figure 6C). Then, a RNA immunoprecipitation (RIP) experiment was used to reversely verify that ACACA can bind to FASRL (Figure 6D). RNA fluorescence in situ hybridization (FISH) and immunofluorescence were used to further confirm the colocalization of the lncRNA FASRL and the ACACA protein (Figure 6E). Subsequently, we determined whether FASRL affects the expression of ACACA. The Western blotting results showed that knocking down FASRL expression did not affect the total protein level of ACACA (Figure 6F).

Previous studies have shown that the phosphorylation of ACACA (Ser79) inhibits its enzyme activity and reduces FA synthesis.^[^ ^23^ ^]^ Interestingly, we found that knocking down FASRL expression increased the level of phosphorylated ACACA (Ser79) (Figure 6F). In addition, we found that FASRL was unable to bind to phosphorylated ACACA (Figure 6C), suggesting that FASRL bound to ACACA and then inhibited its phosphorylation. Notably, ACACA, as a key rate‐limiting enzyme in FA synthesis, is an important protein in FA metabolism and can catalyze acetyl‐CoA to form malonyl‐CoA.^[^ ^24^ ^]^ The ACACA enzymatic activity (Figure 6G,H) and malonyl‐CoA content (Figure 6I,J) were decreased in the HCC cells with FASRL knockdown. To detect the effect of the FASRL–ACACA interaction on the content of FAs, we constructed stable shACACA‐transfected HepG2 cell line to detect their FAs content (Figure S14, Supporting Information). Compared with the control shNC group, we found that several types of FAs were downregulated in both the shFASRL and shACACA groups (Figure 6K). The above results suggest that FASRL bound to ACACA and inhibited its phosphorylation, thus increasing FA synthesis.

### 2.7 FASRL Knockdown Decelerated Lipid Accumulation In vitro and In vivo

**Figure 7:**
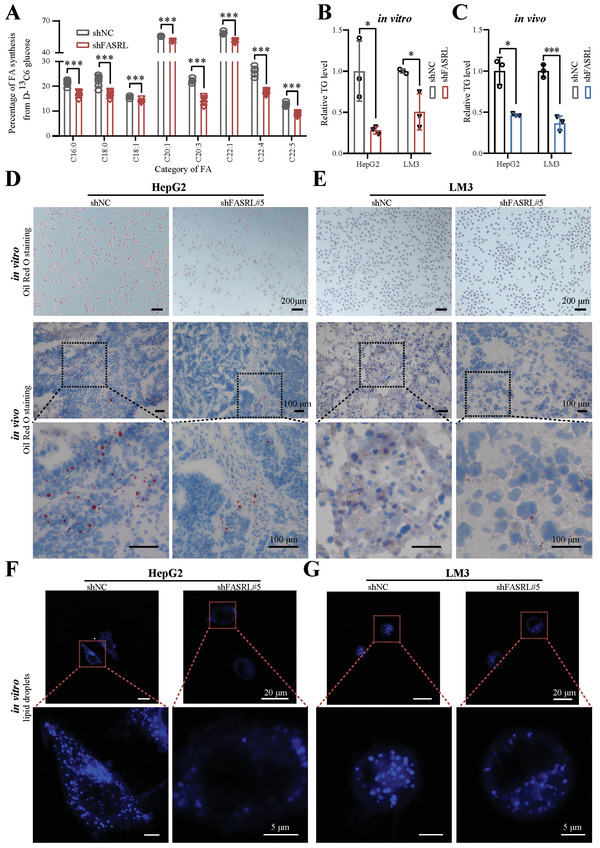
FASRL knockdown suppressed lipid accumulation in HCC in vitro and in vivo. A) Fatty acid (FA) synthesis in D‐glucose‐^13^C_6_‐labeled HepG2 cells stably transfected with shFASRL and its control shNC. The horizontal axis showing different types of FAs, *n* = 6. B,C) The contents of triglyceride (TG) in the shFASRL group and control shNC group in in vitro cells and in vivo models of HCC. D,E) Oil red O staining showing the lipid droplet content in the shFASRL group and control shNC group in vitro cells and in vivo models of HCC. Lipid droplet fluorescence staining showing the lipid droplet content in the shFASRL group and control shNC group in the F) HepG2 and G) LM3 cell lines. The data are expressed as the mean ± SD. *p < 0.05; ***p < 0.001.

Interestingly, consistent with the RNA pulldown results, the FA metabolism pathway was enriched by the FASRL knockdown according to our RNA‐seq data, highlighting the importance of FASRL in lipid metabolism in HCC. Lipids in tumors can provide abundant nutrients for tumor cell growth. Therefore, we sought to further explore whether FASRL could promote the accumulation of lipids in tumor cells. First, we used the stable isotope ^13^C_6_‐labeled glucose to trace FA metabolism and found that after the knockdown of FASRL, newly synthesized FAs labeled with ^13^C were significantly reduced (**Figure** **7**A), further suggesting that FASRL interference inhibited de novo FA synthesis. In both HCC cells and HCC tissues, the FASRL knockdown significantly decreased the triglyceride (TG) content (Figure 7B,C). In addition, no differences in either low density lipoprotein (LDL) or high density lipoprotein (HDL) were observed between the FASRL knockdown and control groups (Figure S15A–D, Supporting Information), suggesting that the change in ACACA enzymatic activity did not affect acetyl‐CoA utilization by non‐FA synthesis. Oil red O staining of HCC cells and xenograft tissues was performed to detect lipid droplets, and the results showed that the lipid droplets were significantly reduced in both HCC cell lines and the corresponding xenograft tumors after FASRL expression was knocked down (Figure 7D,E and Figure S16, Supporting Information). Fluorescent labeling of lipid droplets further verified that the number of lipid droplets in HCC cells was significantly reduced after FASRL expression was knocked down (Figure 7F,G, Supporting Information). In summary, both in vivo and in vitro, knocking down FASRL expression can reduce the accumulation of neutral lipids in HCC cells, which is an important reason for the slower growth of HCC cells.

### 2.8 The Expression of FASRL, USF1, and ACACA Was Increased, and Their High Expression Indicated a Worse Prognosis in HCC Patients

**Figure 8:**
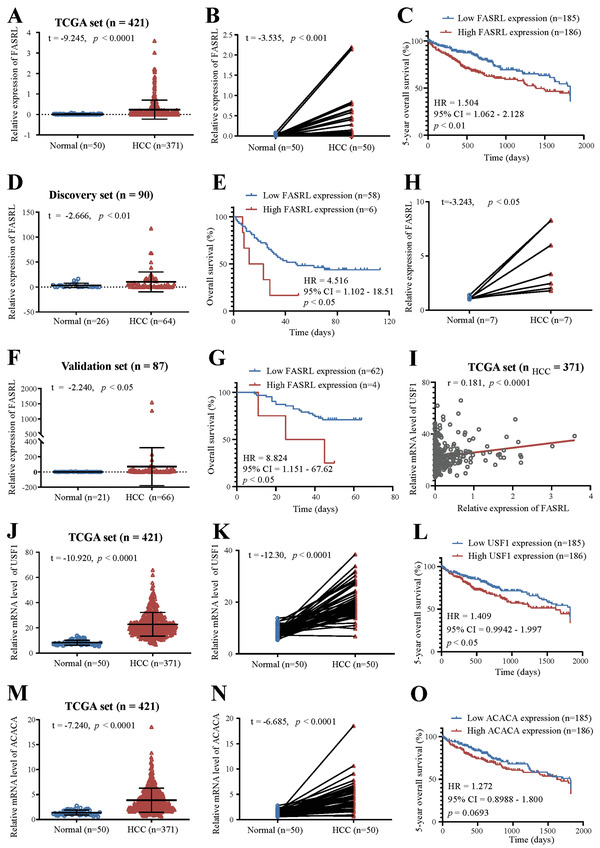
The expression of FASRL, USF1, and ACACA was increased, and their high expression was correlated with a worse prognosis in HCC patients. In the TCGA dataset, A) the relative expression of FASRL in HCC samples and normal liver samples, B) the relative expression of FASRL in paired HCC samples and adjacent samples, and C) the prognosis of HCC patients with high and low FASRL expression. D–G) Based on our discovery set and validation set, D,F) the relative expression of FASRL in HCC samples and normal liver samples, and E,G) the prognosis of HCC patients with high and low FASRL expression. H) FASRL expression in fresh HCC samples and their paired adjacent normal tissues. I) Correlation analysis of the relative expression of USF1 and FASRL in HCC tumor samples. The expression of USF1 and FASRL in HCC tissues was downloaded from TCGA website. *n* for HCC = 371. Based on the TCGA dataset, J) the mRNA level of USF1 in HCC samples and normal liver samples, K) the USF1 mRNA level in HCC samples and corresponding paired adjacent samples, and L) the prognosis of HCC patients with high or low USF1 expression. Based on the TCGA dataset, M) the relative mRNA level of ACACA in HCC samples and normal liver samples, N) the relative mRNA level of ACACA in paired HCC samples and adjacent samples, and O) the prognosis of HCC patients with high and low ACACA expression. The values of *n* and *p* are provided in the panel. The data are expressed as the mean ± SD.

Subsequently, we explored the expression of FASRL in human HCC samples and found that FASRL expression was significantly upregulated in HCC tissues compared with that in normal liver tissues (**Figure** **8**A). Additionally, FASRL expression was significantly increased in HCC tissues compared with that in paired adjacent normal tissues (Figure 8B). Furthermore, high FASRL expression predicted worse overall survival than low FASRL expression (Figure 8C). Then, we validated the data in two commercial arrays and found that these results were similar to those of the first cohort (Figure 8D–G). Additionally, FASRL expression was higher in fresh HCC samples than that in paired normal tissues (Figure 8H). Similar to the results in HCC tissues, FASRL was more highly expressed in HCC cell lines than that in normal liver cell line (Figure S3B, Supporting Information).

In addition, the expression of the upstream TF USF1 was positively correlated with that of FASRL in HCC samples (Figure 8I). Moreover, USF1 was more highly expressed in HCC tissues than that in normal liver tissues (Figure 8J) and that in paired adjacent normal tissues (Figure 8K). A high expression of USF1 was correlated with a worse prognosis in HCC patients (Figure 8L). Finally, we discovered that ACACA was also more highly expressed in HCC tissues than that in adjacent liver tissues, and its high expression was correlated with a worse prognosis in HCC patients (Figure 8M–O). In summary, these results indicate that FASRL, USF1, and ACACA were all highly expressed in HCC patient samples, and their high expression predicted a worse overall survival in patients.

## 3 Discussion

In the present study, we discovered a new lncRNA, FASRL, whose expression was driven by the upstream TF USF1 via a superenhancer. FASRL expression was upregulated in HCC tissues, and its high expression correlated with a worse overall survival. In vitro, we found that knocking down FASRL expression significantly inhibited proliferation and migration, and promoted apoptosis in HCC cells. Conversely, the overexpression of FASRL promoted proliferation and migration in HCC cells. In vivo, the knockdown of FASRL expression significantly reduced the growth of HCC in nude mice. Mechanistically, we further found that FASRL interacted with the FA synthesis rate‐limiting enzyme ACACA, thus increasing FA synthesis and leading to lipid accumulation.

LncRNAs perform various functions. In the nucleus, lncRNAs can regulate splicing, gene expression in *cis* or in *trans*, and nucleation of subnuclear domains.^[^ ^14^, ^25^ ^]^ In the cytoplasm, lncRNAs can act as miRNA sponges, interact with RBPs, and regulate the translation of specific mRNAs.^[^ ^14^, ^26^ ^]^ Several studies have reported the abnormal expression of lncRNAs in HCC,^[^ ^27^ ^]^ due to aberrant epigenetic regulation to a great extent. As robust epigenetic regulatory elements of genes, superenhancers, which contain a cluster of enhancers, are able to greatly promote the expression of cancer‐promoting genes by interacting with their promoters, thus exacerbating tumor progression. LncRNAs constitute an important class of genes whose expression is specifically driven by aberrant superenhancers in multiple tumors.^[^ ^4^, ^28^ ^]^ Our previous study showed that HCCL5 is a superenhancer‐associated lncRNA regulated by the EMT‐related TF ZEB1, which promotes the EMT process to accelerate malignancy.^[^ ^4^ ^]^ However, studies investigating the mechanism underlying the functions of lncRNAs, especially superenhancer‐associated lncRNAs, are far from sufficient. This work screened and discovered a new lncRNA, FASRL, whose expression was driven by the upstream TF USF1 via a superenhancer.

Binding RBPs to regulate downstream pathways is an important mechanism by which lncRNAs function in cells. We found that FASRL interacted with ACACA and affected the phosphorylation of ACACA (S79). Several other studies have also shown that lncRNAs affect the phosphorylation of the proteins they bind to. For example, highly repetitive satellite III lncRNAs can accelerate the rephosphorylation of SRSF9 under heat stress recovery conditions.^[^ ^29^ ^]^ NF‐KappaB interacting lncRNA (NKILA) can bind to NF‐κB/IκB and inhibit IκB phosphorylation.^[^ ^30^ ^]^ Thus, we hypothesized that FASRL may be recruited as an obstacle of phosphokinases to alter the phosphorylation of the target protein or that FASRL may selectively inhibit protein phosphorylation by binding to the phosphorylation site, which needs to be further confirmed.

ACACA is an important enzyme in lipid metabolism and is the central enzyme that controls de novo FA biosynthesis.^[^ ^24^ ^]^ A review published in 2020 mentioned that lipid metabolism is critical for the occurrence and development of tumors, and targeting lipid metabolism may be a potential therapeutic strategy for tumors.^[^ ^5^ ^]^ Tumor cells naturally consume substantial energy to proliferate rapidly, but this energy is not enough to obtain sufficient lipids from the extracellular space, leading to the reactivation of de novo lipogenesis.^[^ ^31^ ^]^ Our research proves that FASRL increases de novo FA synthesis in HCC cells, accelerates lipid accumulation and contributes to sufficient nutrient levels for HCC development. It is difficult to directly manipulate lipids in tumor cells. In contrast, it would be easier to regulate lipid metabolism by targeting upstream factors, such as FASRL. Therefore, our research is very meaningful considering these aspects.

The phosphorylation of ACACA hinders de novo FA synthesis. It has been reported that liver adipogenesis and liver cancer lesion formation were significantly increased in ACACA (S79) (S212) knock‐in mice, and ACACA phosphorylation by AMPK was prevented.^[^ ^23a^ ^]^ ACACA plays an important role in HCC,^[^ ^23a^ ^]^ prostate cancer,^[^ ^32^ ^]^ non‐small cell lung cancer,^[^ ^23c^ ^]^ and breast cancer^[^ ^33^ ^]^ by promoting adipogenesis. These studies revealed that ACACA and its phosphorylation can be used as drug targets to inhibit tumor growth. The small molecule drug ND‐654, which targets ACACA, was reported to mimic ACACA phosphorylation and inhibit tumor growth, revealing that ND‐654 can serve as a potential drug for the treatment of HCC.^[^ ^23a^ ^]^ Additionally, ND‐646 can inhibit tumor growth by targeting ACACA in non‐small cell lung cancer.^[^ ^23c^ ^]^ In contrast, this discovery may be a novel and easily controlled way to promote ACACA phosphorylation. Thus, downregulating the expression of FASRL or its upstream TF may reduce the binding of FASRL to ACACA, thus decelerating HCC progression.

As previously mentioned, this work found that USF1 can drive FASRL expression via a superenhancer. Consistent with a previous report showing that USF1 increases liver lipid accumulation,^[^ ^34^ ^]^ the present work showed that the increased FA biosynthesis due to FASRL was driven by USF1, which is another mechanism by which USF1 increases the lipid contents. Thus, superenhancer‐associated FASRL expression driven by USF1 promoted tumor progression through the interaction between FASRL and ACACA to increase FA synthesis. Therefore, we are likely to develop drugs that inhibit the TF USF1 or superenhancers to reduce the expression of FASRL or inhibit the binding of FASRL and ACACA to reduce FA synthesis and ultimately achieve the goal of inhibiting HCC.

In summary, we identified a new FA synthesis‐related lncRNA, FASRL, whose expression is driven by the TF USF1 through a superenhancer. FASRL increases de novo FA biosynthesis by interacting with ACACA and then leads to lipid accumulation to exacerbate HCC progression (Figure S17, Supporting Information). In addition, FASRL can be used as a potential target for inhibiting HCC.

## 4 Experimental Section

### Human HCC Cell Lines

The HepG2 cell line was purchased from BlueFBio Biology Technology Development Corporation (Shanghai, China), and the LM3 and LO2 cell lines were purchased from FuHeng Biology Corporation (Shanghai, China). The Huh7 and PLC cell lines were kept in our laboratory. These five cell lines were cultured in DMEM (GIBCO, C11995500BT) or 1640 (GIBCO, C11875500BT) supplemented with 10% fetal bovine serum (Biological Industries, 04‐001‐1ACS) in an incubator at 37 °C in 5% CO_2_. The cell lines were verified by a short tandem repeat analysis.

### Chromatin Immunoprecipitation Sequencing and ChIP–qPCR

For ChIP‐seq or ChIP–qPCR, ≈100 mg samples of tissues were sliced and then dispersed adequately with a hand disperser (Kinematica AG, POLYTRON^TM^ PT1200E). Dispersed tissues or 1 × 10^7^ fresh PLC cells were crosslinked with 1% formaldehyde for 45 or 10 min, respectively, neutralized with 125 mm glycine at room temperature, and then sonicated with a Bioruptor Plus sonicator (Diagenode, Belgium) at 4 °C. Sheared chromatin was incubated with 10 µg H3K27ac antibody (Abcam, ab4729) and Protein A/G magnetic beads (Pierce, 88803) with rotation at 4 °C overnight. The samples were washed and decrosslinked at 65 °C overnight.

For ChIP‐seq, purified DNA was used to construct a library and then sequenced by BGI Genomics (Shenzhen, China). For ChIP–qPCR of H3K27ac (Abcam, ab4729) and USF1 (Santa Cruz Biotechnology, sc‐390027X), purified DNA was used for quantitative RT–PCR (qRT–PCR). The primers used for qRT–PCR are shown in Table S3, Supporting Information.

The raw H3K27ac ChIP‐seq data from HepG2 cells were downloaded from the ENCODE database, and those from Huh7 cell line and our previous study with the LM3 cell line were downloaded from the Gene Expression Omnibus (GEO) database. Our raw data and the downloaded raw data were checked by FastQC, aligned to the genome by Bowtie, and peak‐called by using MACS. The rank ordering of superenhancers (ROSE) algorithm (https://bitbucket.org/young_computation/rose) was used to calculate and identify superenhancers, and IGV was used to visualize the identified peaks. The accession information of downloaded sequencing data is shown in Table S4, Supporting Information.

### Chromatin Interaction Analysis by Paired End Labeling Data Analysis

The original sequencing data from a ChIA‐PET in the HepG2 cell line were downloaded from the ENCODE database as previously described.^[^ ^4^ ^]^ In brief, the ChIA‐PET sequencing data of two biological replicates were merged and processed, including linker trimming, read alignment, duplicate removal, peak calling, and chromatin loop calling through ChIA‐PET2.^[^ ^35^ ^]^ Quality control of the ChIA‐PET data was performed by Trimmomatic‐0.33. Finally, WashU Epigenome Browser (http://epigenomegateway.wustl.edu/browser/) was used to generate the identified interaction circle.

### Cell Nuclear and Cytoplasmic Separation

The nuclear and cytoplasmic separation was conducted by an AmbionTM PARIS Kit (Thermo Fisher Scientific, AM1921). In brief, up to 1 × 10^7^ freshly cultured HepG2 and LM3 cells were collected and washed twice with precooled PBS. The cells were then resuspended in 500 µL ice‐cold Cell Fractionation Buffer and incubated for 10 min on ice. Then, the cells were centrifuged at 500 g at 4 °C for 5 min, the cytoplasm was carefully aspirated from the supernatant, and the nucleus was lysed by using Cell Disruption Buffer from the pellet. Finally, the cytoplasmic lysates and nuclear lysates were used for RNA isolation.

### RNA Extraction and qRT–PCR Analysis

According to the manufacturer's instructions, the total RNA was extracted with an RNA Quick Purification Kit (ESscience, RN001). Using the PrimeScript RT Reagent Kit with gDNA Eraser (Takara Bio, RR047A), cDNA was obtained from 1 µg RNA by reverse transcription. A LightCycler 480 SYBR Green I Master Mix (Roche, 4887352001) was used for qRT–PCR to evaluate the RNA expression level. The primer sequences used for qRT–PCR are shown in Table S3, Supporting Information.

### Western Blotting

For the Western blotting, total cell lysates were prepared by extraction in RIPA buffer supplemented with 1% protease inhibitor (Bimake, B14002) and 1% phosphatase inhibitor (Bimake, B15002). The cell lysates were boiled at 100 °C for 10 min, separated by SDS‐polyacrylamide gel electrophoresis (PAGE) and transferred to PVDF membranes. Then, the membranes were blocked with 5% skim milk. Immunoblotting was performed with the corresponding primary antibodies. After incubation with HRP‐conjugated secondary antibodies, Western blot images were captured on a SmartChemi^TM^ 610 (Beijing SinSage Technology Co., Ltd., China). The following primary antibodies were used: TP53INP2 (1:1000; Abcam, ab273 012), ALDH3A1 (1:1000; Abcam, ab129 022), IDI1 (1:1000; Abcam, ab205617), DLD (1:1000; Abcam, ab133551), HMGCS1 (1:500; Proteintech, 17643‐1‐AP), CPOX (1:1000; Abcam, ab169766), ACACA (1:5000; Abcam, ab109368), USF1 (1:10 000; Abcam, ab125020), p‐ACACA (1:1000; Cell Signaling Technology, 11818T), β‐tubulin (1:2000, Beyotime Biotechnology, AF1216) and β‐actin (1:3000; TransGen Biotech, HC201‐01).

### Cell Proliferation Assay

In total, 2.5 × 10^3^ HepG2 cells and 1.5 × 10^3^ LM3 cells per well were seeded in a 96‐well plate with 100 µL of medium supplemented with 1% fetal bovine serum (Biological Industries, 04‐001‐1ACS). A CCK‐8 assay kit (Dojindo, CK04) was used to measure cell proliferation every 2 days for a total of three times.

### EdU Assay

An EdU Cell Proliferation Kit with Alexa Fluor 488 (Epizyme Biomedical Technology, CX002) was used to assess cell proliferation ability. HCC cells (1 × 10^5^) were seeded into individual wells. Then, they were incubated with 10 µm EdU buffer at 37 °C for 2 h, fixed with 4% formaldehyde for 15 min and permeabilized with 0.1% Triton X‐100 for 15 min. Click reaction solution was added to the wells and was followed by the addition of Hoechst 33342 to stain the nucleus. A Leica ultrahigh‐resolution microscope (Leica SP8 STED 3X, Germany) was used for imaging.

### Small Interfering RNA Transfection

The HepG2 and LM3 cell lines were transfected with siRNA to knock down gene expression. The sequences of the siRNAs are listed in Table S5, Supporting Information. siRNAs were transfected into HCC cells using Lipofectamine RNAiMAX Transfection Reagent (Invitrogen, 13778150) according to the manufacturer's instructions. The volume ratio of 20 µm siRNA and RNAiMAX was 1:1. Then, RNA was harvested from the cells 48 h after the siRNA transfection, and protein was harvested from the cells 72 h after the siRNA transfection.

### Construction and Transfection of shRNA Vectors

The shRNA plasmids targeting FASRL (shFASRL#1 and shFASRL#5), USF1 (shUSF1), ACACA (shACACA), and one scramble control shRNA plasmid (shNC) were constructed in the pLKO.1 shRNA expression vector, respectively. The above plasmids were transfected into 293T cells with polyethyleneimine (PEI; Polysciences, 23966‐1G) along with the lentiviral packaging plasmids psPAX2 and pMD2.G. The produced lentiviruses that were released into the supernatant of the cultured 293T cells were then collected and condensed. These condensed lentiviruses were added to HCC cell lines with the viral‐transducing enhancer hexadimethrine bromide (Beyotime, C0351). Finally, positive cells transfected with shFASRL, shUSF1, shACACA, or shNC were selected with puromycin (Solarbio, P8230). The sequences for the vector construction are provided in Table S6, Supporting Information.

### Construction of FASRL‐Overexpressing Stable Cell Lines

The full‐length sequence of FASRL was cloned into the pcDNA3.1 vector. Next, 1 µg pcDNA3.1‐ FASRL overexpression vectors or corresponding empty vectors were transfected into cells that had been cultured in a 6‐well plate with 3 µL of ViaFect Transfection Reagent (Promega, E4982). After transfection for 48 h, G418 (0.7 mg mL^−1^ for HepG2 cell line, 1 mg mL^−1^ for LM3 cell line) was added to the cell cultures, which were cultured for another 2 weeks or more. Stably expressing cells were selected for subsequent experiments.

### Transwell Migration Assay

In total, 8 × 10^4^ HepG2 cells or 4 × 10^4^ LM3 cells were added to a top chamber in serum‐free DMEM. DMEM supplemented with 20% FBS was added to the bottom chamber. The top chamber was stained with 0.1% crystal violet for 10 min and washed with ddH_2_O. A microscope (Nikon NI‐U, Japan) was used to capture the migrated cells on the lower surface of the membrane in the top chamber.

### Nude Mouse Tumor Xenograft Assay

The protocol for the nude mouse tumor xenograft assay was approved by the Institutional Animal Care and Use Committee of Sun Yat‐sen University, and followed the animal ethics and welfare guidelines (2021000060). Briefly, 5 × 10^6^ HepG2 cells and 1 × 10^6^ LM3 cells stably transfected with shFASRL or shNC vectors were subcutaneously injected into 4‐week‐old BALB/c male nude mice (eight nude mice per group). The tumor sizes were measured with a Vernier caliper. The tumor size was calculated by using the following formula: tumor volume (mm^3^) = length (mm) × width^2^ (mm^2^)/2. To comply with the tumor size required by the animal ethics, 1000 mm^3^ of tumor volume was used as a surrogate end point for survival to generate Kaplan–Meier curves. After the mice in each group were sacrificed by cervical dislocation, the xenograft tumors were extracted, imaged, and weighed. The tumors were then fixed in 4% paraformaldehyde and embedded in paraffin for H&E and Ki‐67 staining. Image J 1.52a was used to calculate the intensity and area of Ki‐67 staining for evaluating the proliferation of tumor cells in xenograft tumors.

### Dual‐Luciferase Reporter Assay

Five components (E1‐E5) of the superenhancer of FASRL and negative control (NC) were separately inserted into a pGL3‐promoter vector. The sequences of the enhancer are shown in Table S7, Supporting Information. HepG2 and LM3 cells were seeded in 6‐well culture dishes. The cells were cotransfected with 2 µg vectors (pGL3‐promoter, NC, E1, E2, E3, E4, or E5) and 0.2 µg pRL‐TK (for normalization) by using 6 µL ViaFect Transfection Reagent (Promega, E4982). After transfection for 48 h, a Dual‐Luciferase Reporter Assay System (Promega, E1910) was used to measure the luciferase activities. The ratio of firefly luciferase activity to Renilla luciferase activity was standardized to determine enhancer activity.

### RNA Fluorescence In Situ Hybridization and Immunofluorescence Analysis

RNA FISH was performed in HCC cells using the Ribo Fluorescent In Situ Hybridization Kit (RIBOBIO, China) according to the kit's manual. For immunofluorescence, the cells were fixed with 4% paraformaldehyde, permeabilized with 0.5% Triton X‐100 and blocked with 5% BSA. Then, the cells were incubated overnight with primary antibodies at 4 °C and then with fluorescent secondary antibodies at room temperature. The nucleus was stained with DAPI (Beyotime, C1002). An LSM 880 confocal laser scanning microscope (Zeiss LSM 800 with Airyscan, Germany) was used to photograph the fluorescence signals.

### RNA Pull‐Down Assay and Mass Spectrometry

The full‐length sense and antisense sequences of the FASRL fragment were cloned into the pcDNA3.1 vector using the HindIII restriction enzyme (Thermo Fisher Scientific, FD0504) and XhoI restriction enzyme (Thermo Fisher Scientific, FD0694). A TranscriptAid T7 High Yield Transcription Kit (Thermo Fisher Scientific, K0441) was used to transcribe the full‐length sense and antisense RNA sequences of FASRL in vitro, and then, these sequences were purified using a GeneJET RNA Purification Kit (Thermo Fisher Scientific, K0731). A Pierce RNA 3′ End Desthiobiotinylation Kit (Thermo Fisher Scientific, 20163) was used to label the purified sense and antisense RNA sequences of FASRL with desthiobiotin at the 3′ end. Next, the lncRNA–protein complexes were purified by a Pierce Magnetic Pull‐Down Kit (Thermo Fisher Scientific, 20164) and separated by SDS‐PAGE. The separated proteins were stained with a Fast Silver Stain Kit (Beyotime, P0017S), and then, MS was performed to identify the specific proteins that bound to the FASRL. Finally, immunoblotting was used to verify that the proteins interacted with FASRL.

### RNA Immunoprecipitation Assay

The RIP assay was performed using a Magna RIP RNA‐Binding Protein Immunoprecipitation Kit (Merck Millipore, 17–700) according to the manufacturer's instruction. ACACA antibody (Proteintech, 21923‐1‐AP) or Normal Rabbit IgG antibody (Cell Signaling Technology, 2729S) were incubated with protein A/G magnetic beads (MCE, HY‐K0202‐1 mL) for 1 h at room temperature and then used to precipitate RNA targets from total HepG2 cell lysates. The precipitated RNA was assessed by qRT–PCR to measure the amount of FASRL. The primers used for qRT–PCR are shown in Table S3, Supporting Information.

### RNA‐Seq Analysis and Gene Set Enrichment Analysis

Total RNA was extracted from the HepG2 and LM3 cell lines by using the TRIzol method and then reverse transcribed into double‐stranded cDNA. The double‐stranded cDNA was denatured and circularized by the splint oligo sequence to generate the final library. The RNA‐seq libraries were prepared for sequencing at BGI Genomics (Shenzhen, China).

For RNA‐seq analysis, the raw sequencing data were filtered with SOAPnuke (v1.5.2)^[^ ^36^ ^]^ by removing adapters, low‐quality bases, and unknown bases to obtain clean data in FASTQ format and then mapped to the reference genome using HISAT2 (v2.0.4).^[^ ^37^ ^]^ The clean reads were aligned to the reference coding gene set with Bowtie2 (v2.2.5),^[^ ^38^ ^]^ and the expression level of the gene was computed with RSEM (v1.2.12).^[^ ^39^ ^]^

Differential expression analysis was performed using DESeq2 (v1.4.5)^[^ ^40^ ^]^ with a *Q* value corrected with a rigorous threshold (*Q* value ≤ 0.05) by Bonferroni correction. The *Q* value was adjusted for multiple testing using the procedure of Benjamini and Hochberg. A heatmap was constructed with the package pheatmap (v1.0.8) according to the gene expression in different samples.

GSEA^[^ ^41^ ^]^ was applied to statistically identify differences in the hallmark biological pathways between the two groups. A GSEA was performed using the online GSEA software provided by BGI‐genomics, which uses predefined gene sets from the Molecular Signatures Database (MSigDB v5.0). In the present study, we used HALLMARK gene sets from MSigDB for GSEA and a list of genes ranked based on a score calculated as log10 of the *p*‐value multiplied by the sign of the fold change. The minimum and maximum criteria for the selection of gene sets from the collection were 10 and 500 genes, respectively.

### Oil Red O Staining

Tissues or cells were fixed with 4% paraformaldehyde for 10 min, washed for 5 min, and then dyed with Oil red O dye solution for 10–15 min (the Oil red O dye solution was prepared by mixing Oil red O saturated solution with distilled water at a ratio of 6:4). The tissues were washed with 60% isopropanol for 1 min. For the cells, this washing step was omitted. The tissues or cells were washed with distilled water, counterstained with Mayer hematoxylin, washed with distilled water for 1–3 min, and then covered with glycerine gelatin. A microscope (Nikon NI‐U, Japan) was used to capture images.

### Triglyceride Quantification

HCC xenografts from nude mice or cell lines were fully lysed in absolute ethanol, and the TG concentrations were measured with a Triglyceride Assay Kit (Nanjing Jiancheng Bioengineering Institute, A110‐1‐1). The data were normalized to the tumor weights or the cell concentrations as appropriate.

### Lipid Droplet Fluorescence Detection

The lipid droplet fluorescence detection was performed with a Lipid Droplet Assays Kit‐Blue (Dojindo Laboratories, LD05). Briefly, HepG2 cells were washed twice with HBSS. After the working solution was added to the cells, the cells were incubated in a 37 °C, 5% CO_2_ incubator for 2 h. Then, the cells were washed twice with HBSS, and images were captured by a Leica ultrahigh‐resolution microscope (Leica SP8 STED 3X, Germany).

### ACACA Enzymatic Activity Assay

The ACACA enzyme activity assay was performed according to the protocol of the Human ACC ELISA Kit (AIDISHENG, ADS‐W‐ZF016‐96). HCC cells (5 × 10^6^) were added to 1 mL extraction solution and disrupted with sonication on ice. The supernatant was obtained after centrifugation of the mixture at 12 000 rpm and 4 °C for 10 min. The protein concentration (*Cpr*) was measured via a BCA Protein Assay Kit (CWBIO, CW0014). The supernatant (10 µL) was added to a 96‐well plate, and reagent 1, reagent 2, reagent 3, and reagent 4 from the kit were sequentially added. After incubation at 37 °C for 10 min, reagent 5 was added, and the contents were mixed. The absorbance was measured immediately at 340 nm (value *A1*) and after 10 min at 340 nm (value *A2*) using a microplate reader (Tecan, Spark10M). ACACA enzymatic activity (nmol/min/mg prot) = [Δ*A* ÷ (*ε* × *d*) × *V*2 × 10^9^] ÷ (*V*1 × *Cpr*) ÷ *T* = 643.1 × Δ*A* ÷ *Cpr*, where Δ*A* = *A*1 − *A*2; *ε* is the NADH molar extinction coefficient, 6.22 × 10^3^ L mol^−1^ cm^−1^; *d* is the light diameter of the 96‐well plate, 0.5 cm; *V*1 is the sample volume, 0.01 mL; *V*2 is the total volume of reaction system, 0.2 mL; and *T* is the reaction time, 10 min.

### Determination of Malonyl‐CoA, Low Density Lipoprotein, and High Density Lipoprotein Content by ELISA

Malonyl‐CoA, LDL, and HDL contents were measured by ELISA kits (malonyl‐CoA: Meimian, MM‐51489H1; LDL: Meimian, MM‐1209H1; HDL: Meimian, MM‐1247H1). Cells were lysed by sonication with 200 W power, 3 s sonication, and 10 s intervals 30 times on ice. The lysates were centrifuged at 12 000 rpm and 4 °C for 10 min to obtain the supernatant. The protein concentration of the supernatant was measured with a BCA Protein Assay Kit (CWBIO, CW0014). Supernatant with a fivefold dilution was added to ELISA plates precoated with specific antibodies. After incubation of the supernatant at 37 °C for 30 min, 50 µL of enzyme labeling reagent was added and reacted with 50 µL chromogenic reagent A and 50 µL chromogenic reagent B. Reactions were terminated by adding 50 µL stop reagent, and the absorbance at 450 nm was measured within 15 min using a microplate reader (Tecan, Spark10M).

### Determination of Fatty Acids by Gas Chromatography‐Mass Spectrometry Analysis

HepG2 cells stably transfected with shFASRL, shACACA, or shNC were harvested, and FAs were extracted. Gas chromatography‐mass spectrometry (GC‐MS) analysis was performed by BioNovoGene (Suzhou, China).

### Determination of Glucose Incorporation into De Novo Fatty Acid Synthesis by Liquid Chromatography‐Mass Spectrometry Analysis

HepG2 cells (5 × 10^6^) were washed twice with PBS and incubated with 10 mL culture medium containing 10% FBS, 90% glucose‐free DMEM, and 25 mm d‐glucose‐^13^C_6_ (MCE, HY‐B0389A) for 24 h. After the culture medium was removed, the cells were washed twice with precooled PBS, mixed with 1 mL precooled methanol, and collected via scraping for liquid chromatography‐mass spectrometry (LC‐MS) analysis. For LC‐MS analysis, an ACQUITY UPLC HSS T3 column (Waters, Ireland) was used for reversed‐phase chromatographic analyses. An ultra‐performance liquid chromatography system (Agilent Technologies, Agilent 1290 II) coupled to a quadrupole‐TOF MS instrument (AB SCIEX, 5600 Triple TOF Plus) was applied to acquire metabolome data. The information‐dependent acquisition mode was applied for MS/MS analyses of the metabolites. Data acquisition and processing were performed with Analyst^TM^ TF 1.7.1 software (AB SCIEX, Canada).

### Clinical Samples

HCC fresh tissues were collected from Sun Yat‐Sen Memorial Hospital (Guangzhou, China) with written informed consent from patients, and the procedures were approved by the Institutional Review Board of the Sun Yat‐sen Memorial Hospital of Sun Yat‐sen University (SHY JS‐CP‐1707013). HCC specimens (seven tumor samples and seven matching samples of adjacent normal tissues) were obtained from patients, quickly processed to ensure the quality of the clinical samples, and stored at −80 °C. Commercial HCC and tumor‐adjacent liver tissue cDNA arrays (HLivH090Su01 and HLivH087Su02) were purchased from OUTDO (Shanghai Outdo Biotech Company, China) and were approved by the Ethics Committee of Shanghai Outdo Biotech Company. The discovery set (HLivH090Su01) contains 64 cancer tissues and 26 adjacent nontumorous liver tissues from HCC patients. The validation set (HLivH087Su02) contains 66 cancer tissues and 21 adjacent nontumorous liver tissues from HCC patients. Survival data in the discovery set and validation set were provided by OUTDO (Shanghai Outdo Biotech Company, China). In addition, the RNA‐seq and clinical data, including survival information of the 371 of HCC samples (no metastases) and RNA‐seq data of 50 adjacent normal liver samples, were downloaded from the TCGA website (https://cancergenome.nih.gov/).

### Survival Analysis

In TCGA set, according to the median expression of FASRL, USF1, and ACACA, HCC patients were divided into high and low expression groups. In discovery set and validation set, according to the best cutoff value of FASRL expression determined by using X‐tile software,^[^ ^42^ ^]^ the HCC patients were divided into the following two groups: high and low FASRL expression groups. Overall survival was defined as the time from the date of surgery to death or last follow up. Kaplan–Meier curve was constructed for overall survival analysis using a Gehan–Breslow–Wilcoxon. All survival‐related plots were generated using GraphPad Prism 8.0.1 software.

### Statistical Analysis

All analyses were performed with SPSS 17.0. All the values were expressed as the mean ± SD. All experiments with cell lines were repeated at least three times, and representative data were shown. For comparisons between two groups, the data were first checked for homogeneity of variance and the significance of differences was determined by Student's *t*‐test. A one‐way analysis of variance (ANOVA) with proper post‐hoc tests was used for the comparisons among three or more groups. Significance is indicated as follows: NS, non‐significance; *p < 0.05; **p < 0.01; ***p < 0.001. The value of *n* in each legend represents the number of repetitions or the sample size. ImageJ 1.52a was used for the quantification of histology. The *p*‐values, *n* values, and precision measures are provided in the corresponding figure legends.

## Conflict of Interest

The authors declare no conflict of interest.

## Authors Contribution

Study concept and design: L.P.; acquisition of data: J.‐Y.P., D.‐K.C., R.‐L.Z., and C.‐Y.Z.; analysis and interpretation of data: J.‐Y.P. and L.P.; drafting of the manuscript: J.‐Y.P.; critical revision of the manuscript for important intellectual content: L.P., X.‐Q.Y., and S.‐F.C.; statistical analysis: L.P.; obtained funding: L.P., S.‐F.C., and X.‐Q.Y.; administrative, technical, or material support: L.P. and G.‐C. L.; study supervision: L.P. and X.‐Q.Y.

## Supporting information

Supporting Information

## Data Availability

The data that support the findings of this study are available from the corresponding author upon reasonable request. The raw sequencing data generated in this study are available in NCBI GEO under the accession number GSE206549. The H3K27ac ChIP‐seq data of PLC cells are stored in GEO under the accession number GSE206547; the RNA‐seq data of HepG2 and LM3 cells transfected with siFASRL or siNC are stored in GEO under the accession number GSE206548.
