# Supplementary material for: Upregulation of Superenhancer‐Driven LncRNA FASRL by USF1 Promotes De Novo Fatty Acid Biosynthesis to Exacerbate Hepatocellular Carcinoma
Source: Adv Sci (Weinh). 2022 Oct 28;10(1):2204711. doi: 10.1002/advs.202204711 (PMC9811444; doi:10.1002/advs.202204711)
Supplement: Supplementary file 1 — Supporting Information [file ADVS-10-2204711-s001.pdf]

ADVANCED SCIENCE
Open Access

Supporting Information

for *Adv. Sci.*, DOI 10.1002/advs.202204711

Upregulation of Superenhancer-Driven LncRNA FASRL by USF1 Promotes De Novo Fatty Acid Biosynthesis to Exacerbate Hepatocellular Carcinoma

*Jiang-Yun Peng, Dian-Kui Cai, Ren-Li Zeng, Chao-Yang Zhang, Guan-Cheng Li, Si-Fan Chen, Xiao-Qing Yuan and Li Peng**

Supporting Information

Upregulation of Superenhancer-driven LncRNA FASRL by USF1 Promotes *De Novo* Fatty Acid Biosynthesis to Exacerbate Hepatocellular Carcinoma

Jiang-Yun Peng, Dian-Kui Cai, Ren-Li Zeng, Chao-Yang Zhang, Guan-Cheng Li, Si-Fan Chen, Xiao-Qing Yuan, Li Peng[*]

Supporting Figures

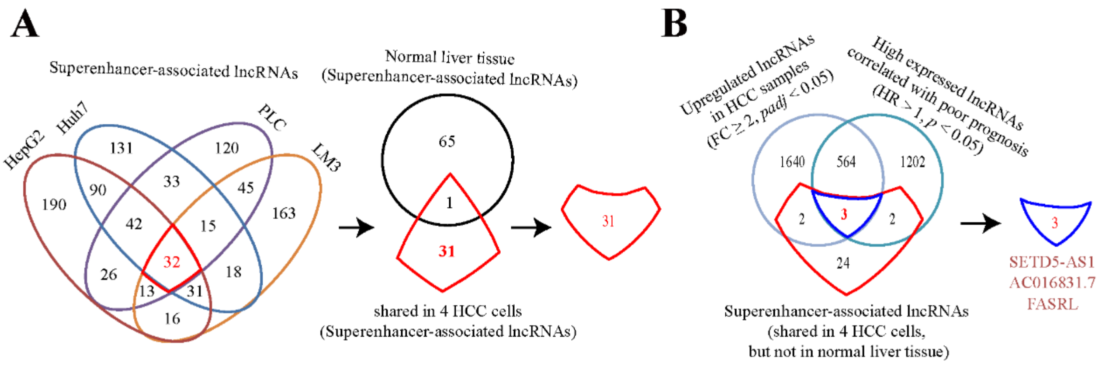

Figure S1. The screening process of superenhancer-associated lncRNAs with potential clinical value in HCC. (A) Venn diagram showing the superenhancer-associated lncRNAs shared by HepG2, Huh7, PLC and LM3 cells, excluding those in normal liver tissues. (B) Venn diagram exhibiting 3 superenhancer-associated lncRNAs, SETD5-AS1, AC016831.7 and FASRL, in the HCC cell lines that were upregulated in the HCC samples and their high expression was correlated with a worse prognosis.

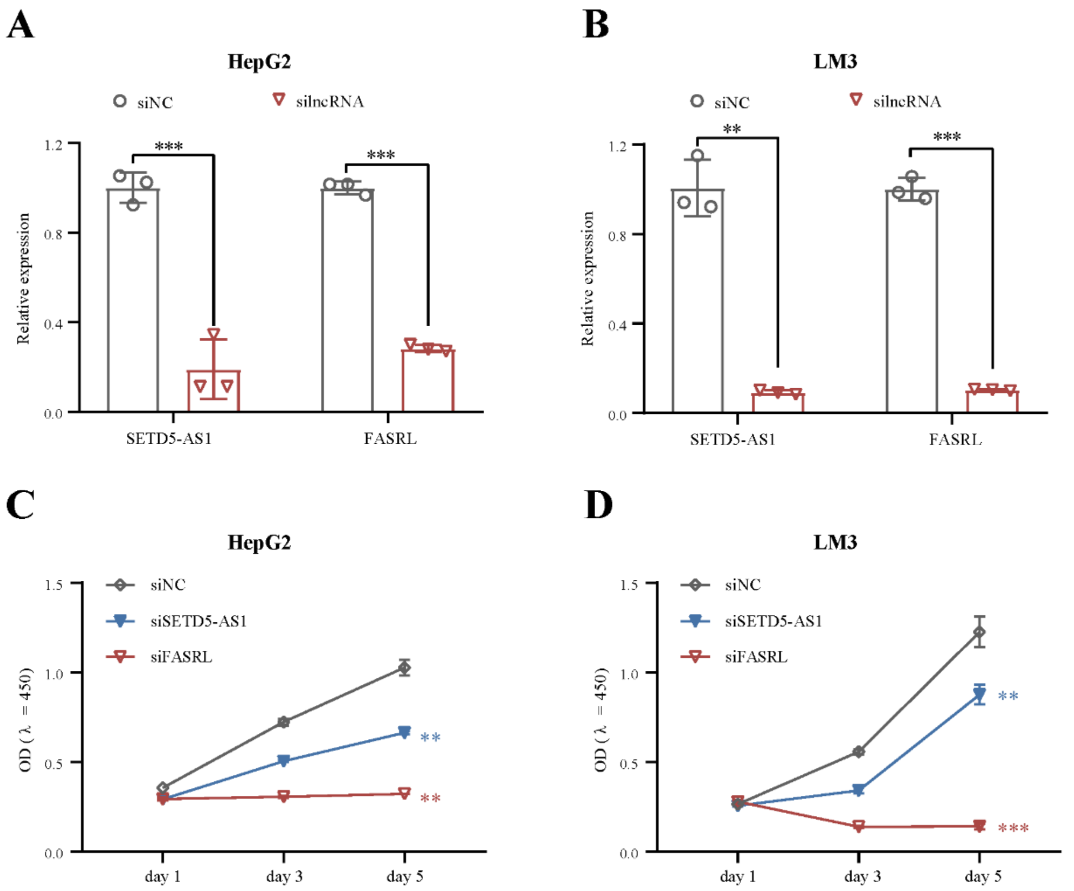

Figure S2. Effect of siRNA-mediated SETD5-AS1 and FASRL interference on cell proliferation in HCC cell lines. (A, B) qRT–PCR assay detecting the knockdown effect on the (A) HepG2 and (B) LM3 cell lines transfected with siRNA pools targeting SETD5-AS1 and FASRL. (C, D) CCK-8 assay showing the proliferation of the (C) HepG2 and (D) LM3 cell lines transfected with siSETD5-AS1, siFASRL and siNC. The data are expressed as the mean ± SD. **, p<0.01; ***, p<0.001

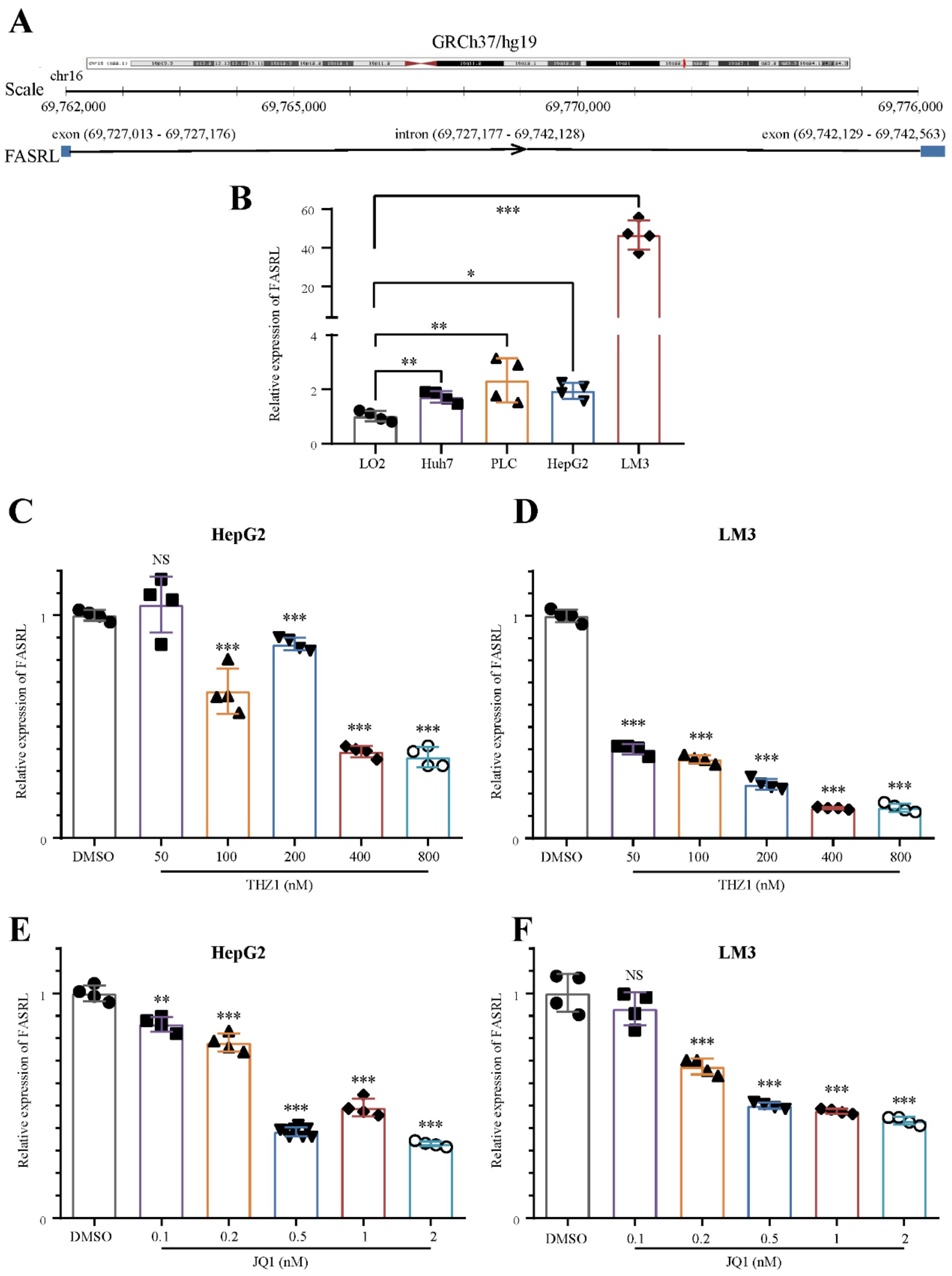

Figure S3. A novel superenhancer-related lncRNA, FASRL, was identified in HCC. (A) The location of FASRL in the genome, and the arrow indicates the FASRL transcription direction. (B) The relative expression of FASRL in the normal liver cell line LO2 and four HCC cell lines, Huh7, PLC, HepG2 and LM3. (C, D) The relative expression of FASRL in the (C) HepG2 and (D) LM3

cell lines treated with the CDK7 inhibitor THZ1. (E, F) The relative expression of FASRL in the (E) HepG2 and (F) LM3 cell lines treated with the BRD4 inhibitor JQ1. The data are expressed as the mean ± SD. NS, non-significance; *, p<0.05; **, p<0.01; ***, p<0.001

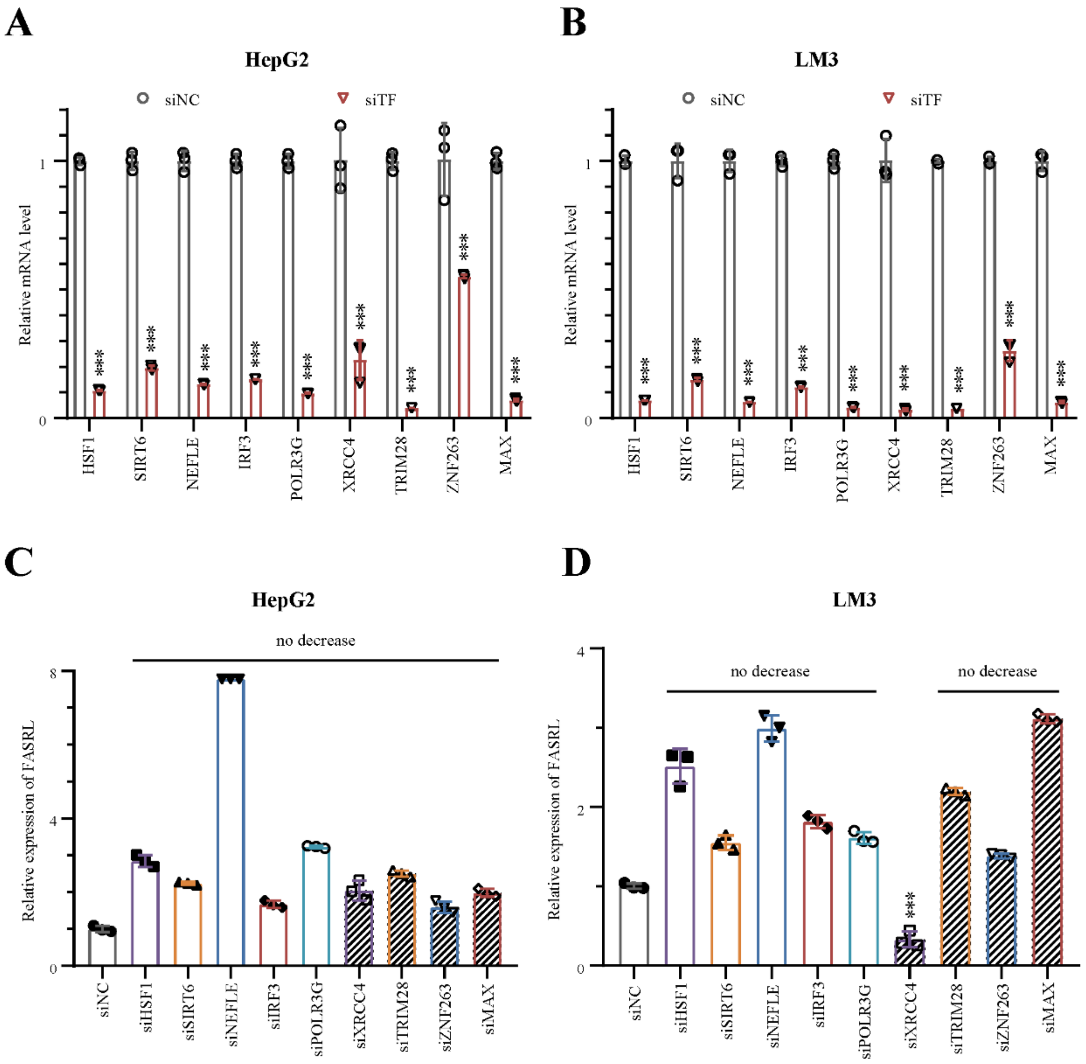

Figure S4. Screening for the upstream transcription factors of FASRL. (A, B) qRT–PCR assay showing the efficiency of the siRNA pool-mediated knockdown of 9 TFs in the (A) HepG2 and (B) LM3 cell lines. (C, D) qRT–PCR showing the expression of FASRL in the (C) HepG2 and (D) LM3 cell lines transfected with siRNA pool-mediated knockdown of 9 TFs. The data are expressed as the mean ± SD. ***, p<0.001

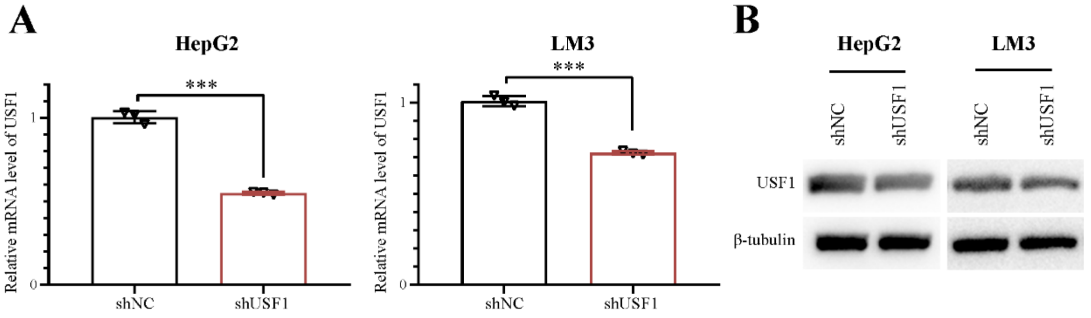

Figure S5. Knockdown efficiency of USF1 shRNA in HCC cell lines. (A) qRT–PCR assay showing the mRNA level of USF1 in the (left) HepG2 and (right) LM3 cell lines with shRNA-mediated USF1 knockdown. (B) Western blot assay showing the protein content of USF1 in the (left) HepG2 and (right) LM3 cell lines after the shRNA-mediated USF1 knockdown. The data are expressed as the mean ± SD. ***, p<0.001

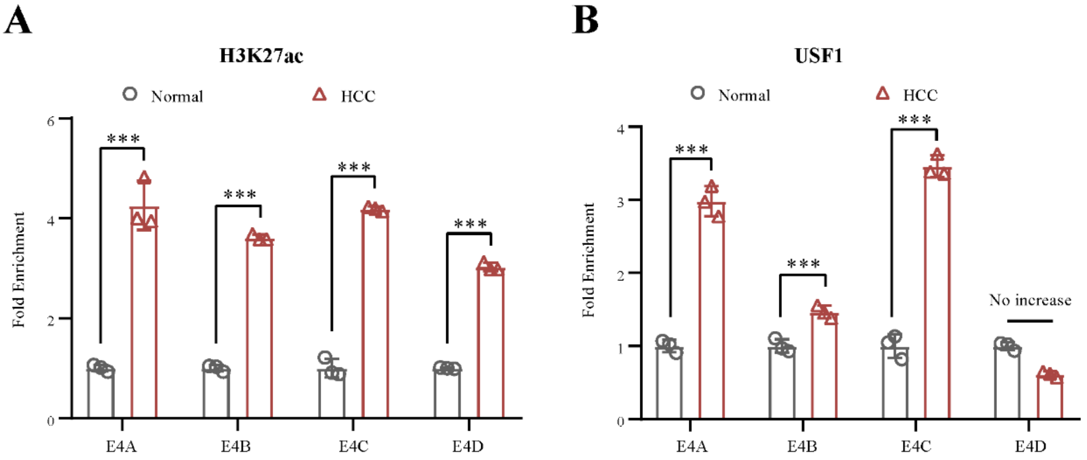

Figure S6. ChIP–qPCR of H3K27ac and USF1 in HCC patient tissues. (A) ChIP–qPCR of H3K27ac of the superenhancer components in tumor and paired normal liver tissues from HCC patients. (B) ChIP–qPCR of USF1 of the superenhancer components in tumor and paired normal liver tissues from HCC patients. The data are expressed as the mean ± SD. ***, p<0.001

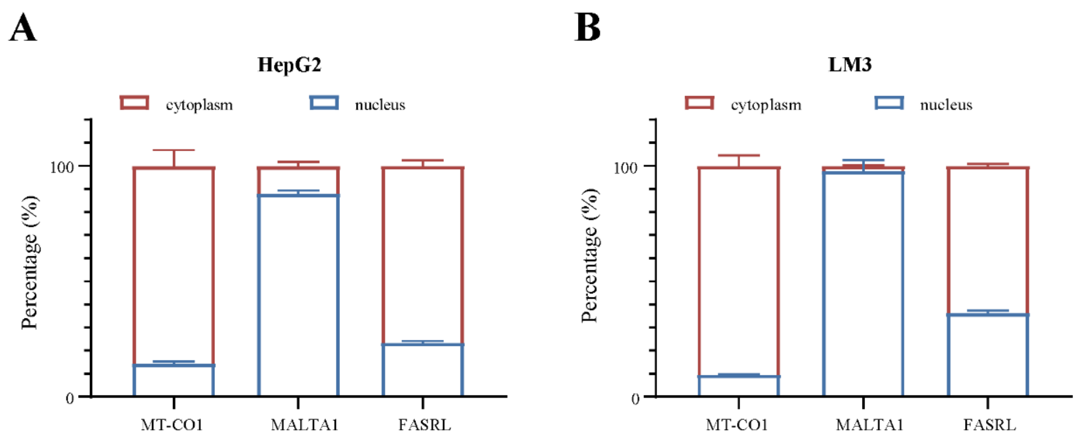

Figure S7. Subcellular distribution of FASRL. (A, B) FASRL was distributed in the cytoplasm and nucleus in the (A) HepG2 and (B) LM3 cell lines. MT-CO1 is a marker of the cell cytoplasm, and MALAT1 is a marker of the cell nucleus. The data are expressed as the mean ± SD.

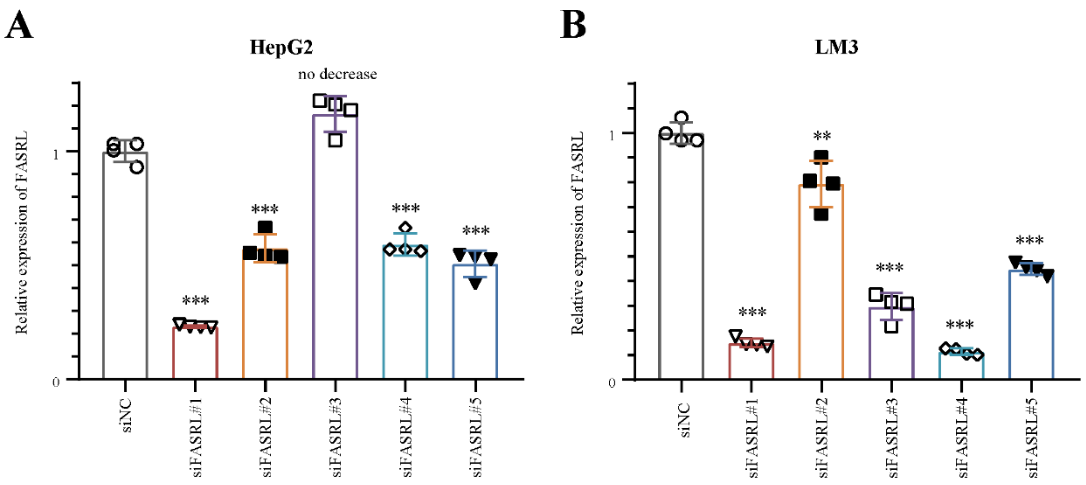

Figure S8. Interference effect of siRNAs targeting FASRL in HCC cells. (A, B) The effects of 5 different siRNA sequences specifically targeting FASRL on FASRL expression in (A) HepG2 and (B) LM3 cell lines. The data are expressed as the mean ± SD. **, $p<0.01$; ***, $p<0.001$

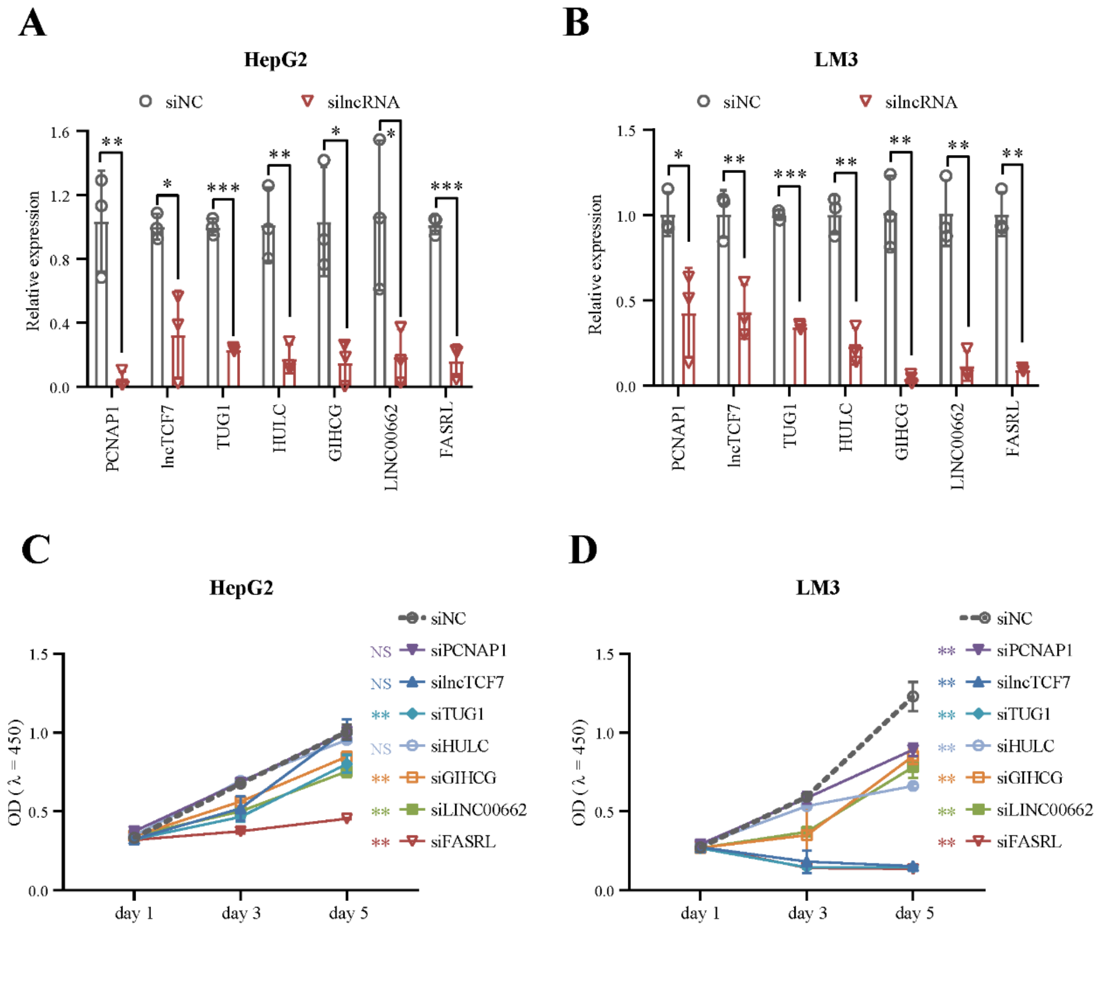

Figure S9. The effect of FASRL knockdown on cell proliferation in HCC cell lines. (A, B) The knockdown effect of lncRNA PCNAP1, lncTCF7, TUG1, HULC, GIHCG, LINC00662 and FARSL in the (A) HepG2 and (B) LM3 cell lines transfected with the corresponding siRNA pools. (C, D) CCK-8 assay revealing the effect of the siRNA pool-mediated knockdown of lncRNA PCNAP1, lncTCF7, TUG1, HULC, GIHCG, LINC00662 and FARSL on cell growth in the (C) HepG2 and (D) LM3 cell lines. The data are expressed as the mean ± SD; NS, non-significance; *, p<0.05; **, p<0.01; ***, p<0.001

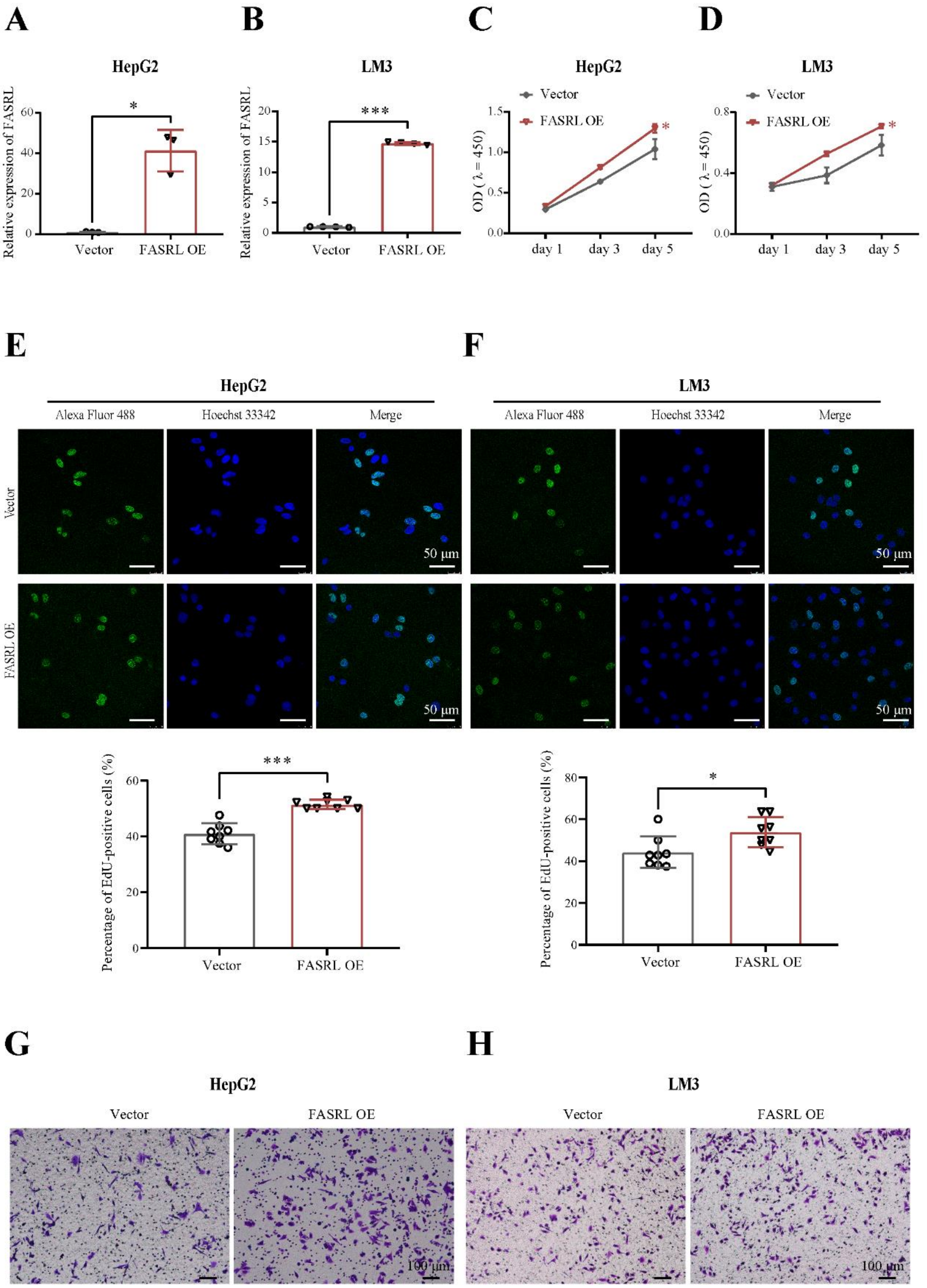

Figure S10. The effect of FASRL overexpression on the proliferation and migration of HCC cell lines. (A, B) The FARSL overexpression in (A) HepG2 and (B) LM3 cell lines. (C, D) CCK-8 assay revealing the growth of the (C) HepG2 and (D) LM3 cell lines with FARSL overexpression or its control. (E, F) EdU assay revealing the proliferation of (E) HepG2 and (F) LM3 cell lines

with FARSL overexpression or its control. (G, H) Transwell assay showing the cell migration ability of the (G) HepG2 and (H) LM3 cell lines with FARSL overexpression or its control, n=8 fields. OE, overexpression. The data are expressed as the mean ± SD. *, p<0.05; ***, p<0.001

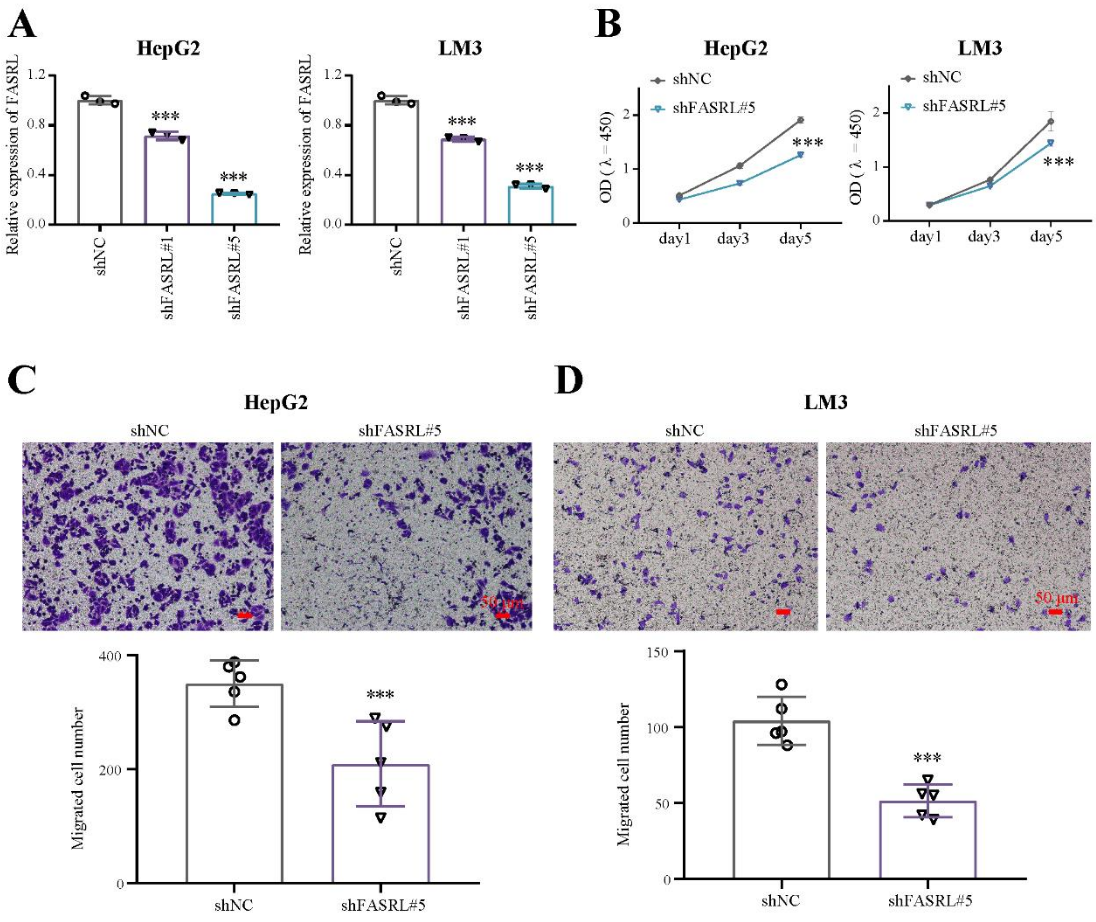

Figure S11. The effect of shRNA-mediated FASRL knockdown on HCC progression *in vitro*. (A) The relative expression of FASRL in the (A) HepG2 and (B) LM3 cell lines stably transfected with shFASRL#1, shFASRL#5 and control shNC. (B) CCK-8 assay showing the growth of the HepG2 and LM3 cell lines stably transfected with shFASRL#5 and control shNC. (C, D) Upper panel, the cell migration ability of the (C) HepG2 and (D) LM3 cell lines stably transfected with shFASRL#5 and control shNC as determined by a Transwell assay. Bottom, the corresponding statistical data of the results in the upper panel. The data are expressed as the mean ± SD. ***, p<0.001

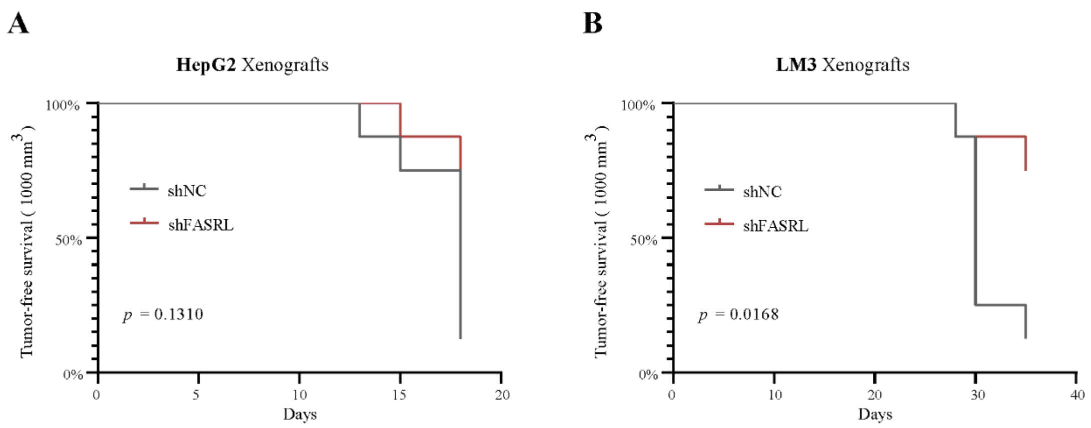

Figure S12. The effect of FASRL knockdown on HCC survival in nude mice. (A, B) Kaplan–Meier survival curves of the shFASRL group and control shNC group of HCC xenograft tumors derived from the (A) HepG2 and (B) LM3 cell lines, *n*=8.

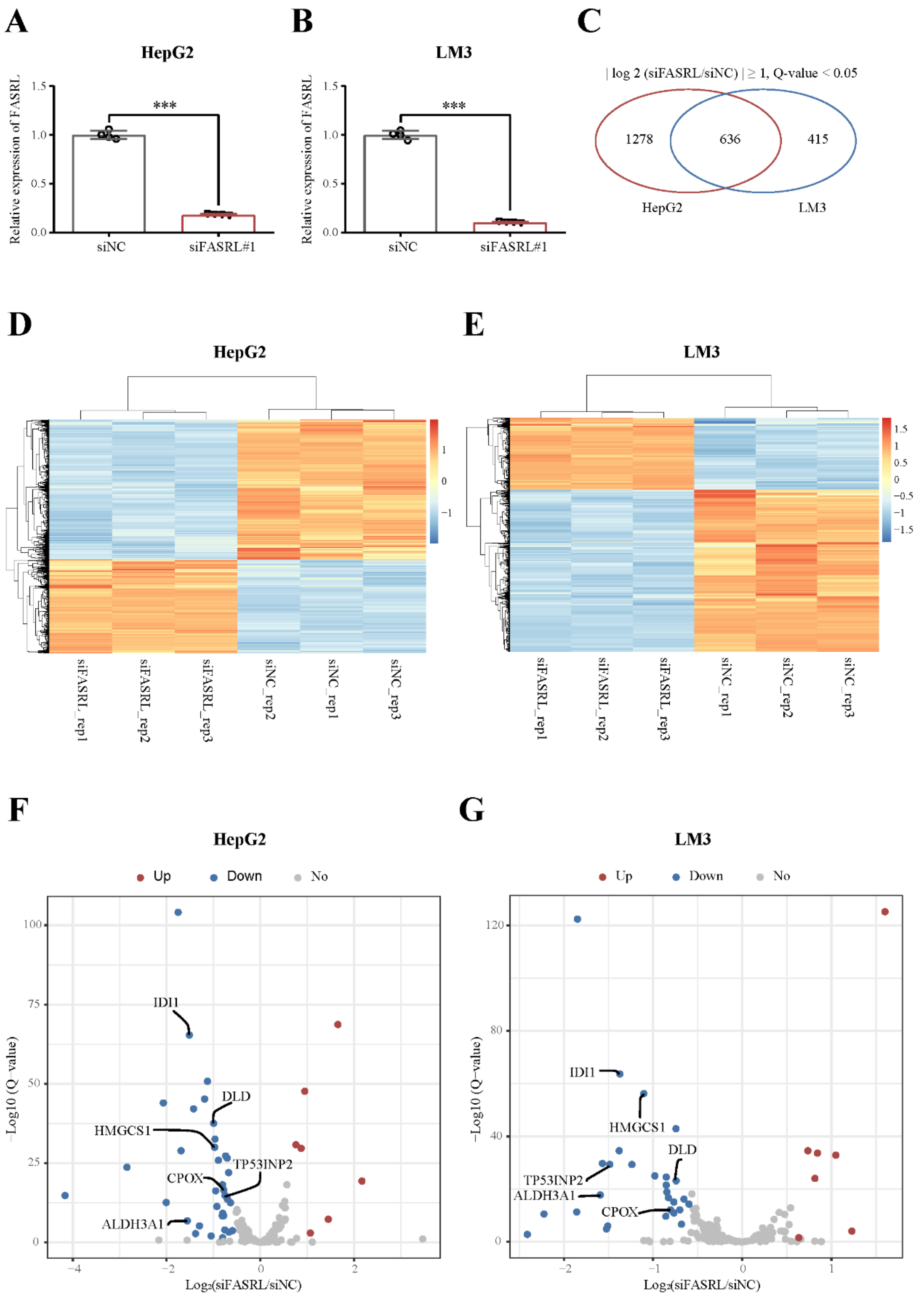

Figure S13. RNA-seq analysis after the knockdown of FASRL in HCC cell lines. (A, B) The knockdown effect of siRNA targeting FASRL in the (A) HepG2 and (B) LM3 cell lines. (C) The Venn diagram showing a total of 636 differentially expressed genes with |log2 (siFASRL/siNC)|≥1

and Q value<0.05 that were shared in the HepG2 and LM3 cell lines after FASRL knockdown. (D, E) Heatmap showing the cluster analysis results of genes with altered expression in (D) HepG2 and (E) LM3 cell lines after FASRL knockdown. (F, G) Volcano plot showing the upregulated genes (red dots), downregulated genes (blue dots) and genes with unchanged expression (gray dots) in fatty acid metabolism pathway in (F) HepG2 and (G) LM3 cell lines after FASRL knockdown. Up, upregulated; Down, downregulated. The data are expressed as the mean ± SD. ***, p<0.001

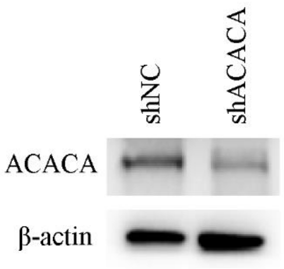

Figure S14. Knockdown efficiency of ACACA shRNA in HCC cell line. Western blot assay showing the protein content of ACACA in the HepG2 cell line after the shRNA-mediated ACACA knockdown.

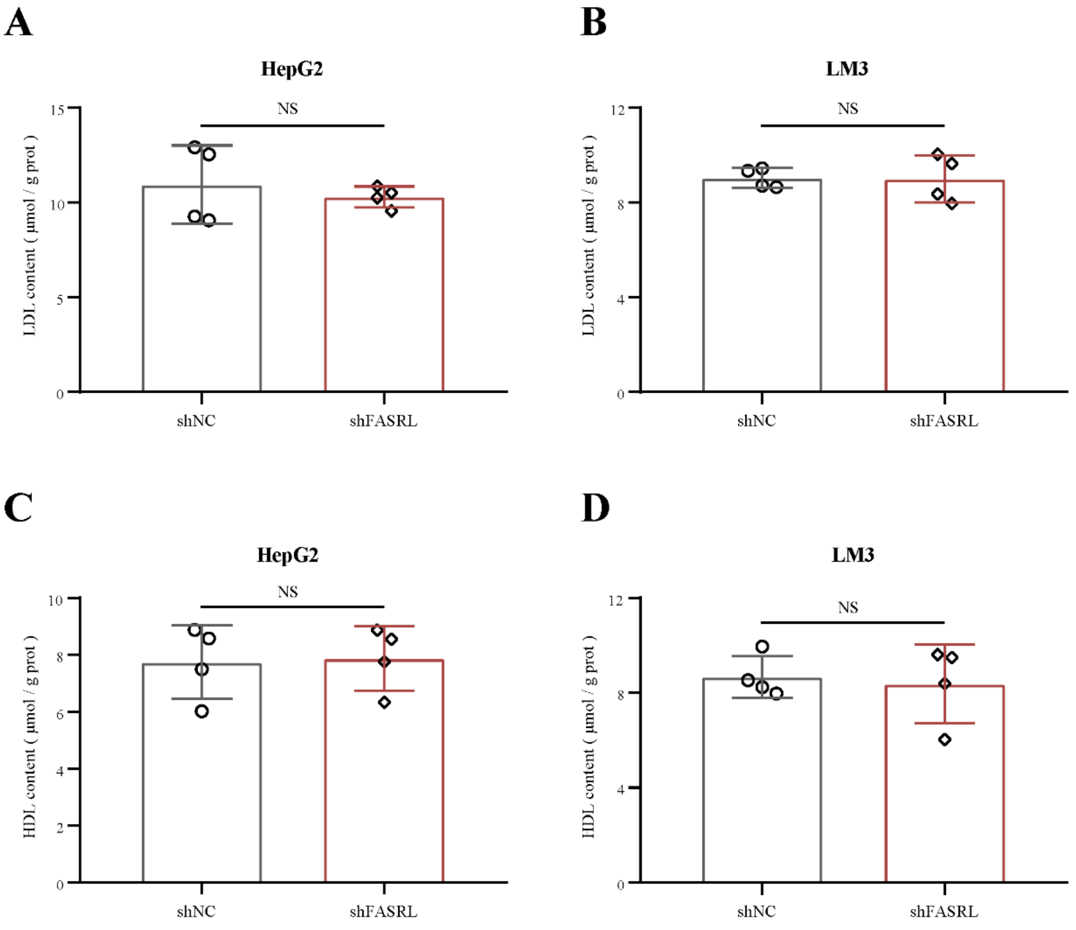

Figure S15. The effect of FASRL knockdown on the LDL and HDL contents in HCC cell lines. (A, B) LDL levels in (A) HepG2 and (B) LM3 cells stably transfected with shFASRL and control shNC, *n*=4. (C, D) HDL levels in (C) HepG2 and (D) LM3 cells stably transfected with shFASRL and control shNC, *n*=4. The data are expressed as the mean ± SD. NS, non-significance

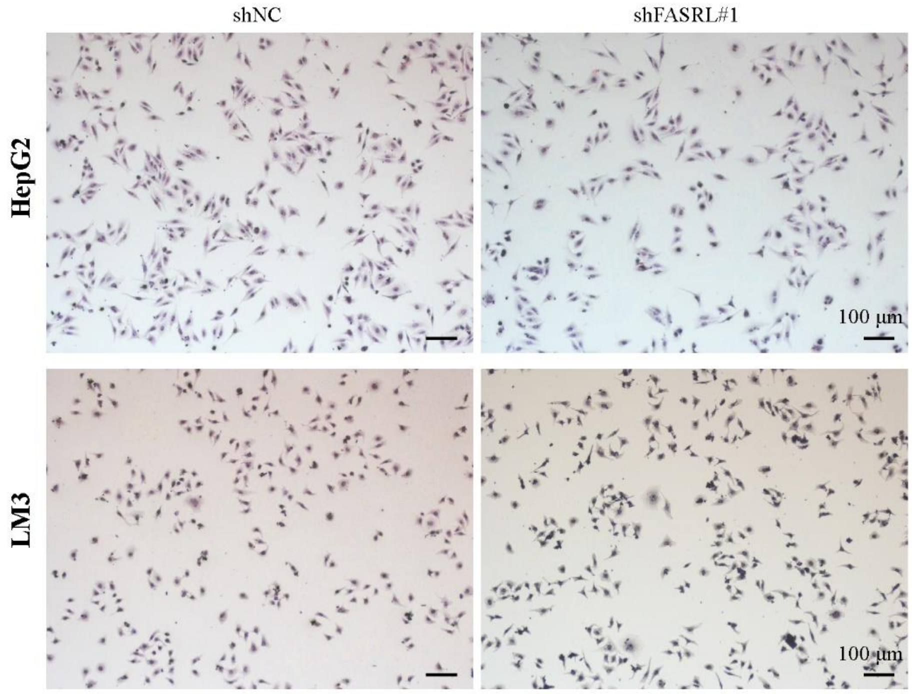

Figure S16. The effect of FASRL knockdown on lipid droplets in HCC cell lines. (A) Oil red O staining showing the lipid droplet content in the shFASRL#1 group and control shNC group in (upper) HepG2 and (bottom) LM3 cell lines.

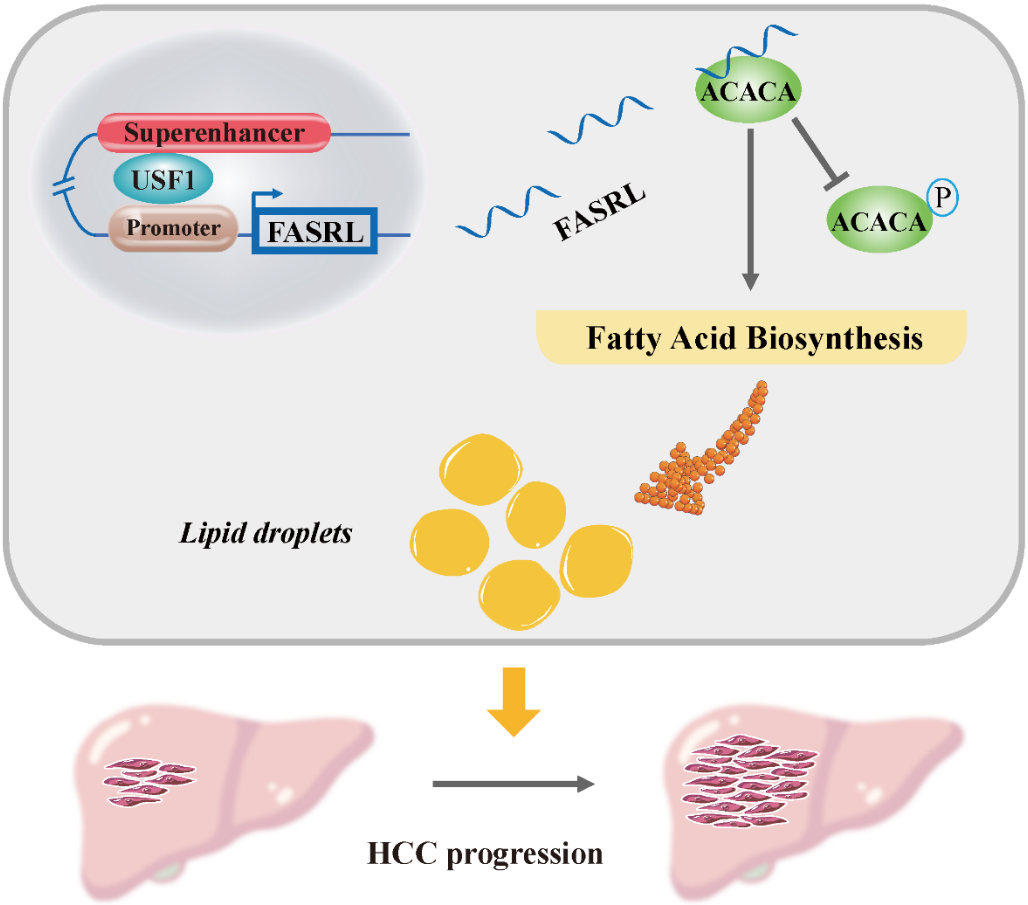

Figure S17. Graphical overview. USF1 binds at the promoter and superenhancer regions of FASRL to upregulate FASRL expression, which inhibits the phosphorylation of ACACA by the binding of FASRL to ACACA. This leads to an increase in *de novo* fatty acid synthesis and lipid accumulation, thus promoting HCC development.

Supporting Tables

Table S1. GSEA of RNA-seq data in HepG2 cells with siRNA-mediated FASRL interference

NAME	MSigDB H(hallmark) Term Desc	ES	NES	NOM p-val	FDR q-val
HALLMARK_BILE_ACID_METABOLISM	Genes involve in metabolism of bile acids and salts.	-0.53329	-1.89798	0	0
HALLMARK_COAGULATION	Genes encoding components of blood coagulation system; also up-regulated in platelets.	-0.47883	-1.73183	0	0.002771
HALLMARK_CHOLESTEROL_HOMEOSTASIS	Genes involved in cholesterol homeostasis.	-0.48431	-1.63571	0.001164	0.007763
HALLMARK_FATTY_ACID_METABOLISM	Genes encoding proteins involved in metabolism of fatty acids.	-0.42791	-1.56587	0	0.014919
HALLMARK_KRAS_SIGNALING_DN	Genes down-regulated by KRAS activation.	-0.3975	-1.46788	0.00105	0.045156
HALLMARK_PEROXISOME	Genes encoding components of peroxisome.	-0.40777	-1.44068	0.007726	0.051031
HALLMARK_XENOBIOTIC_METABOLISM	Genes encoding proteins involved in processing of drugs and other xenobiotics.	-0.37322	-1.40133	0.007292	0.071562
HALLMARK_ESTROGEN_RESPONSE_LATE	Genes defining late response to estrogen.	-0.36401	-1.35743	0.013528	0.103394

Table S2. GSEA of RNA-seq data in LM3 cells with siRNA-mediated FASRL interference

NAME	MSigDB H(hallmark) Term Desc	ES	NES	NOM p-val	FDR q-val
HALLMARK_CHOLESTEROL_HOMEOSTASIS	Genes involved in cholesterol homeostasis.	-0.58156	-1.76233	0	8.28E-04
HALLMARK_BILE_ACID_METABOLISM	Genes involve in metabolism of bile acids and salts.	-0.52538	-1.64339	0	0.005273
HALLMARK_APICAL_SURFACE	Genes encoding proteins over-represented on the apical surface of epithelial cells, e.g., important for cell polarity (apical area).	-0.56679	-1.63811	0.002144	0.004164
HALLMARK_INTERFERON_ALPHA_RESPONSE	Genes up-regulated in response to alpha interferon proteins.	-0.5151	-1.61158	0	0.003823
HALLMARK_FATTY_ACID_METABOLISM	Genes encoding proteins involved in metabolism of fatty acids.	-0.49829	-1.57017	0	0.005537
HALLMARK_KRAS_SIGNALING_DN	Genes down-regulated by KRAS activation.	-0.48938	-1.55989	0	0.00594
HALLMARK_ESTROGEN_RESPONSE_LATE	Genes defining late response to estrogen.	-0.47424	-1.50861	0	0.013324
HALLMARK_PEROXISOME	Genes encoding components of peroxisome.	-0.47269	-1.46464	0.00813	0.022892
HALLMARK_INTERFERON_GAMMA_RESPONSE	Genes up-regulated in response to IFNG [GeneID=3458].	-0.45355	-1.4504	0	0.02554
HALLMARK_APICAL_JUNCTI	Genes encoding components of apical	-0.44844	-1.43091	0.001	0.02838

ON	junction complex.				
HALLMARK_XENOBIOTIC_METABOLISM	Genes encoding proteins involved in processing of drugs and other xenobiotics.	-0.44209	-1.42514	0	0.028045
HALLMARK_COAGULATION	Genes encoding components of blood coagulation system; also up-regulated in platelets.	-0.44525	-1.39686	0.008065	0.036904
HALLMARK_MYOGENESIS	Genes involved in development of skeletal muscle (myogenesis).	-0.42119	-1.35342	0.003006	0.057993
HALLMARK_GLYCOLYSIS	Genes encoding proteins involved in glycolysis and gluconeogenesis.	-0.41083	-1.33025	0.006024	0.07305
HALLMARK_ESTROGEN_RESPONSE_EARLY	Genes defining early response to estrogen.	-0.40459	-1.30413	0.013026	0.092804
HALLMARK_ADIPOGENESIS	Genes up-regulated during adipocyte differentiation (adipogenesis).	-0.39696	-1.28479	0.025075	0.109706
HALLMARK_NOTCH_SIGNALING	Genes up-regulated by activation of Notch signaling.	-0.46063	-1.25678	0.162281	0.142596
HALLMARK_COMPLEMENT	Genes encoding components of the complement system, which is part of the innate immune system.	-0.38471	-1.23245	0.042084	0.174151
HALLMARK_MTORC1_SIGNALING	Genes up-regulated through activation of mTORC1 complex.	-0.37652	-1.21526	0.061122	0.197975
HALLMARK_HEDGEHOG_SIGNALING	Genes up-regulated by activation of hedgehog signaling.	-0.42904	-1.20407	0.216667	0.212501
HALLMARK_KRAS_SIGNALI	Genes up-regulated by KRAS activation.	-0.37612	-1.19856	0.081162	0.213797

Table S3. List of the primer pairs used for qRT–PCR and ChIP–qPCR

Gene name	Forward primer (5'-3')	Reverse primer (5'-3')	Application	Manufacturer
GAPDH	CTGGGCTACACTGAGCACC	AAGTGGTCGTTGAGGGCAATG	qRT-PCR	IGE
FASRL	GGCCCATACAACACACTTCC	AAATAACAGGCGGGCTTCAC	qRT-PCR	IGE
SETD5-AS1	ATGAGGTCTTGCTGTGTTGC	GCAACATGGCAAGATCCTGT	qRT-PCR	IGE
HSF1	CCATGAAGCATGAGAATGAGGC	CTTGTTGACGACTTTCTGTTGC	qRT-PCR	IGE
SIRT6	GTGCCAAGTGTAAGACGCAG	CCTTGCCTTAGCCACGGT	qRT-PCR	IGE
NELFE	CAAGGTGGTGTCAAACGCTC	CTTCCCCTCAAGGGTTCGAG	qRT-PCR	IGE
IRF3	GGACTTTTCCCAGCCAGACA	TGGGGCCAACACCATGTTAC	qRT-PCR	IGE
USF1	TCCCAGACTGCTCTATGGAGA	CGGTGGTTACTCTGCCGAAG	qRT-PCR	IGE
POLR3G	TAGGGAGCAGTGCCTTTCAGAAT	CCTCTTCCTTTATTCCCAGCCAT	qRT-PCR	IGE
XRCC4	ATGTTGGTGAACTGAGAAAAGCA	GCAATGGTGTCCAAGCAATAAC	qRT-PCR	IGE
TRIM28	TGAGACCTGTGTAGAGGCG	CGTTCACCATCCCGAGACTT	qRT-PCR	IGE
ZNF263	CAGTTGCCTGAGAGCTTAGAGG	TGACTGGGAATGTGAGACTCC	qRT-PCR	IGE
MAX	CCGAGGTTTCAATCTGCGG	GAGGTCGTCAATATCTTGCTGG	qRT-PCR	IGE
ALDH3A1	CTCGTCATTGGCACCTGGAACT	CTCGCCATGTTCTCACTCAGCT	qRT-PCR	IGE
IDI1	GCCGCAGACTGTGCTCAAAGC	CCTGTTGCTTGTCGAGGTGGTT	qRT-PCR	IGE

DLD	GGGACTAGAAGAGCTGGGAATTG	CATCCTCTGCTTTGTGAGCCAG	qRT-PCR	IGE
HMGCS1	AAGTCACACAAGATGCTACACCG	TCAGCGAAGACATCTGGTGCCA	qRT-PCR	IGE
CPOX	TGAAGGAGGCTTGTGACCAGCA	AGCGAAACACCTCCTCCTTGGA	qRT-PCR	IGE
TP53INP2	TTCGTGTCGGAGGAGGATGAAG	AACCAGCTCTCGTCCATCAAGG	qRT-PCR	IGE
E4A	GGGAGTCGCGTGTGTAGTGC	CCGCCCTTGTAGGCTGTCCA	ChIP-qPCR	IGE
E4B	GACCCAACGCCTGAATCCTG	CCAGCCCCGAGAGACTTTTC	ChIP-qPCR	IGE
E4C	GGGAGGGGATTGTAGGGGTG	CTCTTGGGACGACTTCCACC	ChIP-qPCR	IGE
E4D	AGTCCAGGGATCAAGTGGGCA	CTTCCAAATCCGCAGTCACAG	ChIP-qPCR	IGE

Table S4. Deposited Data table

Deposited Data	Source	Identifier
H3K27ac ChIP-seq in Normal liver tissues	EMBL-EBI (https://www.ebi.ac.uk/)	SRR2002343
H3K27ac ChIP-seq in HepG2 cells	UCSC (http://genome.ucsc.edu/ENCODE/downloads.html)	wgEncodeBroadHistoneHepg2H3k27acStdRawDataRep2
H3K27ac ChIP-seq in Huh7 cells	EMBL-EBI (https://www.ebi.ac.uk/)	SRR4453268
H3K27ac ChIP-seq in PLC cells	This paper	
H3K27ac ChIP-seq in LM3 cells	https://www.ncbi.nlm.nih.gov/geo/query/acc.cgi?acc=GSE181753	GSM5510395
USF1 ChIP-seq in HepG2 cells	https://www.ncbi.nlm.nih.gov/geo/query/acc.cgi?acc=GSE32465	GSM803527
ChIA-PET in human HepG2 cells	ENCODE (https://www.encodeproject.org)	ENCSR146FPM
RNA-seq in HCC cells	CCLE (https://portals.broadinstitute.org/ccle)	CCLE_ID: _LIVER

RNA-seq in TCGA LIHC samples	TCGA (https://cancergenome.nih.gov/)	LIHC
RNA-seq in HepG2 and LM3 cells transfected with siFASRL and siNC	This paper	

Table S5. List of the siRNA sequences of target genes

Gene name	Targets	Sense (5'-3')	Antisense (5'-3')	Application	Manufacturer
FASRL	#1	GCUCCGUGGAG ACGAAUUUTT	AAAUUCGUCUC CACGGAGCTT	siRNA	GenePharma
	#2	GCCAUGGGACA GUCCAGUUTT	AACUGGACUGU CCCAUGGCTT	siRNA	GenePharma
	#3	GCCCGCCUGUU AUUUCCAUTT	AUGGAAAUAAC AGGCGGGCTT	siRNA	GenePharma
	#4	GGCCAGACGCC CAUCCAAUTT	AUUGGAUGGGC GUCUGGCCTT	siRNA	GenePharma
	#5	UCUGGAAGCGU UGGGAUUUTT	AAAUCCCAACG CUUCCAGATT	siRNA	GenePharma
SETD5-AS1	#1	GTGATGACATCC TCATTGATT	TCAATGAGGAT GTCATCACTT	siRNA	GenePharma
	#2	GAATGGTGCTAT ACTTGAATT	TTCAAGTATAGC ACCATTCTT	siRNA	GenePharma
	#3	CAGGACAGTTCT TATGGCTTT	AGCCATAAGAA CTGTCCTGTT	siRNA	GenePharma
HSF1	#1	UGAGAACAUCA AGAGGAAA	UUUCCUCUUGA UGUUCUCA	siRNA	RiboBio
	#2	CAUUCCAUGCC CAAGUAUA	UAUACUUGGGC AUGGAAUG	siRNA	RiboBio
	#3	UCGUCAACAAG CUCAUUCA	UGAAUGAGCUU GUUGACGA	siRNA	RiboBio
SIRT6	#1	AGUCUUCCAGU GUGGUGUU	AACACCACACU GGAAGACU	siRNA	RiboBio
	#2	GGAACAUGUUU GUGGAAGA	UCUUCCACAAA CAUGUUCC	siRNA	RiboBio

	#3	GUAGAAACUGU GGAUUCUU	AAGAAUCCACA GUUUCUAC	siRNA	RiboBio
NELFE	#1	GCCUUCGUCAC CUAUGAAA	UUUCAUAGGUG ACGAAGGC	siRNA	RiboBio
	#2	AAGUCAACAUA GCCCGAAA	UUUCGGGCUAU GUUGACUU	siRNA	RiboBio
	#3	ACCAAGAACUC AGGCUUCA	UGAAGCCUGAG UUCUUGGU	siRNA	RiboBio
IRF3	#1	UGGAGAAUACU GUGGACCU	AGGUCCACAGU AUUCUCCA	siRNA	RiboBio
	#2	CACCACCUCAA CCAAUAAA	UUUAUUGGUUG AGGUGGUG	siRNA	RiboBio
	#3	AGACAUUCUGG AUGAGUUA	UAACUCAUCCA GAAUGUCU	siRNA	RiboBio
USF1	#1	AGACGCACUAU ACUUACUU	AAGUAAGUAUA GUGCGUCU	siRNA	RiboBio
	#2	CGAGACAAGAU CAACAACU	AGUUGUUGAUC UUGUCUCG	siRNA	RiboBio
	#3	GAGAUACAAAG ACCUCCAA	UUGGAGGUCUU UGUAUCUC	siRNA	RiboBio
POLR3 G	#1	GGCUGUUGGAU UUAGCAAA	UUUGCUAAAUC CAACAGCC	siRNA	RiboBio
	#2	UGAAACACCUG AAGAAAGA	UCUUUCUUCAG GUGUUUCA	siRNA	RiboBio
	#3	CCACUCAUCUG GUAUAACU	AGUUAUACCAG AUGAGUGG	siRNA	RiboBio
XRCC4	#1	GCCTGATTCTTC ACTACCT	GCCTGATTCTTC ACTACCT	siRNA	RiboBio
	#2	GCAATCTGTTCT GAAATGA	TCATTTCAGAAC AGATTGC	siRNA	RiboBio
	#3	CACTGATATTGC ACCAAGT	CACTGATATTGC ACCAAGT	siRNA	RiboBio
TRIM28	#1	GCAACAGUGCU UCUCCAAATT	UUUGGAGAAGC ACUGUUGCTT	siRNA	GenePharma
	#3	GGGACAAACAU GCAACAUUTT	AAUGUUGCAUG UUUGUCCCTT	siRNA	GenePharma

	#5	GGAUGACAGUG CCACCAUUTT	AAUGGUGGCAC UGUCAUCCTT	siRNA	GenePharma
ZNF263	#1	GAACCCACGTTT CCTGTCA	GAACCCACGTTT CCTGTCA	siRNA	RiboBio
	#2	GTGCAACATTTG CGGAAAA	TTTTCCGCAAAT GTTGCAC	siRNA	RiboBio
	#3	GGAAGGTGTTCC GTCTGTA	GGAAGGTGTTCC GTCTGTA	siRNA	RiboBio
MAX	#1	CAAAGACAGCU UUCACAGUTT	ACUGUGAAAGC UGUCUUUGTT	siRNA	GenePharma
	#3	GGAGAGCGACG AAGAGCAATT	UUGCUCUUCGU CGCUCUCCTT	siRNA	GenePharma
	#5	GCAGCACCAUC UCUGCCUUTT	AAGGCAGAGAU GGUGCUGCTT	siRNA	GenePharma

Table S6. List of the shRNA sequences

Gene Name	Targets	Sequence (5'-3')	Application	Manufacturer
FASRL	#1	CCGGGCTCCGTGGAGACGAAT TTCTCGAGAAATTCGTCTCCAC GGAGCTTTTTGAATT	shRNA	IGE
	#5	CCGGTCTGGAAGCGTTGGGAT TTCTCGAGAAATCCCAACGCTT CCAGATTTTTGAATT	shRNA	IGE
USF1		CCGGCGAGACAAGATCAACAA CTCTCGAGAGTTGTTGATCTTG TCTCGTTTTTGAATT	shRNA	IGE
ACACA		CCGGTACAAGGGATACAGGTA TTTACTCGAGTAAATACCTGTA TCCCTTGTATTTTTGAATT	shRNA	IGE

Table S7. List of the FASRL enhancer sequences inserted into the pGL3-promoter vector

Enhancer Name	Sequence (5'-3')
NC	CTGAGGCAGGAGAATGGCATGAACCCAGGAAGCGGAGCTTGCAG

TGAGCCGAGATCACGCCACTGCACTCCAGCCTGGGCGACAGAGC
AAGACTCCGTCTCAAAAAAAAAAAAAAAAAAAAAAAAAAAAAA
AGCACAGATGGGGCTGGGCGTGGTGGCTCACACCTGTAATCCCCG
CACTTTGGGAGGCCAAGGTGGGTGGATCACCTGAGGTCAGGAGTT
CAAGACCAGCCTGGGCAGCATGGTGAAAACCAGTCTCTATTTAAA
AAAATAATAATAATACAGAAATTAGTTGGGCACAGCAGCACTTG
CCTGTAATCCCAGCTACTCGGGAGGCTGAGGCAGAAGAA

E1 GCACTCCAGCCTGCGCAACAGAGCAAGCCTCTGTCTCAAACAAAC
AAACAAAAAGATGCTTGTGAATAGGCAATTTAAAAGTTTTCTGGG
CCCTGGAGGGTTCATTTTCCCTTCCTGCCACTTAACTAGCTGAGCA
ACATGACCTGGGAGGAGTTAGACTATTAGAGTTTACCCTGAGAGA
GGCCACAAGATGCACACACGGATAACCTCTTCAGGGTACAATGG
CATTAGTGTGTTAGGAGAGGTAAGGGACAAAGTTCAACAGAAGG
CTGGGTGTGGTGGCTCATGCCTGTAATCGTAGCACTTTGGGAGAC
CGAGGCGGGTGGATTACTTGAGGTCAGGAGCTAGAGACCAGCCT
GGCCAACATGGTGAAACCCC

E2 CATTACCACCGCCCTTTTTTGTGTTTAAGCTACACGTAAATACAGA
ACATTCTGTAAGATTGAAAAGTCAGGAAATAGTTCCTGCCCTAGT
ATTGGAGGAGAGGGTGCCAGTGCCTCGACGCTGAAGAATGAAAT
ACAAGGGAAACAGCAACCACCGTGTATGCGGCAAGCCTCTCAGC
TGTCAGAATCTATTCCCTGCTGTCTCTGCCAGTAACTCATGCTGGG
CTACCAATCCCCAGCTTAGTCCCTTTCTCAGAGATCCTGATAATTT
CAGTTTGTCAACTGCAAGGGCAGTCTGTTCCTCCACTCACTTTGAC
CAAGATGGGCTTCTTTGTGTACAGGCGCATTTTTAGAGGCAGGAC
C

E3 AGCAACTTATTTCAAAGTGTTTTAGATACTATAGACCAAAAGAGG
TATGGGCTGTGACCGTAAGCCTGATTTGGCCTCTGACGGCCAGTT
TCTGACCACAGTTTTGGAGGGAATTTCTGCTTCCAAGGTCAAGTTT
CTCCTAAATGCCCACTAGAGTTCGTGGGGTGGAGTGAGGAAGGAT
CCGCGACACCGGATACAAACAGAAGCTCTCCTTCTCCCGGAGTCC
GATCAAGGCTCATCCCAGGTCCCTAATCTCTTCCCTTTGTGGGTTT
TGAGTCAAGGAACAAAATTCAGGGCCAAGCCCCTACAACCTCCTC
CACAGGCACCAGTGCTCGAGAGACGACCGCCAAGCACCCCGCCC
TTTGCAGCACTCACCGACCATGGCTCTGGTGCAGTCCGGGGCGCT

E4 GGGAGTCGCGTGTGTAGTGCACGGTGCATTCTCTCTTATATATCCT
GTCCGGCCCGTTTGAGGTGGACAGCCTACAAGGGCGGGACCCAA

CGCCTGAATCCTGGGGTGCTGGGCGGAGGATGCTGAGGCTGCAG
CTCCAGGAAGGGAGGGAGCCTGGGGGATGAAGGAGGGGCCTCTC
CACTCTCACCCGCCGGAGCAACTGGTGGAAGTCAAGAAAAGTCTC
TCGGGGCTGGGGGAGGGGATTGTAGGGGTGTGTGTGACAGAGGC
CTCAAAAATCTGGCTGGGGGTGCTAAGGCAAGCCCATCTGCGCAC
TGTGCCCTGAGGTGCAAGAGGATGCAGGGTGGAAGTCGTCCCAA
GAGAGTCCAGGGATCAAGTGGGCATGCCCTTTTAGCCTTGGCACG
AAATGGAGCAGAAAAAGAGCCGGATGCGGATTACTGTGGTGCCC
TAGGCTCAGATTCTGCTGAGTCACTGTGACTGCGGATTTGGAAG

E5 TACAACACACTTCCCTGGGTGCATGCCACTGCACCACCCCTGCTC
CTCAAGGCAGGTAATGGGGGAACTAATTGGTTTTGTTTCCCATGA
GCTAAGGCAAGGCTGGTGTGGAGATAGCAGTTATAGGGAAGCAT
ATTAAAGAATAGCCAAAACGGGACCAGGCGCGGTGGCTCACGCC
TGTAATCCCAGCAATATGGGAGGCCGAGGTGGGTGGATCACCTG
AGGTCAGGAGTTCGAGACCAGCCTGGCCAACCTGGTGAAGCCCC
GTCTGTACTGAAAAATTAGCTGGGCGTGATGGCGGGCGCCTGTAA
GTCCCAGCTACTCGGGAGGCTGAGG
